# Group B *Streptococcus *vaccine development: present status and future considerations, with emphasis on perspectives for low and middle income countries

**DOI:** 10.12688/f1000research.9363.1

**Published:** 2016-09-22

**Authors:** Miwako Kobayashi, Johan Vekemans, Carol J. Baker, Adam J. Ratner, Kirsty Le Doare, Stephanie J. Schrag

**Affiliations:** 1 National Center for Immunization and Respiratory Diseases, Division of Bacterial Diseases, Centers for Disease Control and Prevention, Atlanta, USA; 2 Epidemic Intelligence Service, Centers for Disease Control and Prevention, Atlanta, USA; 3 Initiative for Vaccine Research, World Health Organization, Geneva, Switzerland; 4 Department of Pediatrics, Baylor College of Medicine, Houston, USA; 5 Department of Molecular Virology and Microbiology, Baylor College of Medicine, Houston, USA; 6 Center for Vaccine Awareness and Research, Texas Children's Hospital, Houston, USA; 7 Departments of Pediatrics and Microbiology, New York University School of Medicine, New York, USA; 8 Centre for International Child Health, Imperial College, London, UK

**Keywords:** Group B Streptococcus, neonatal sepsis, maternal vaccination, vaccine development, low- and middle-income countries, phase III trial

## Abstract

Globally, group B *Streptococcus* (GBS) remains the leading cause of sepsis and meningitis in young infants, with its greatest burden in the first 90 days of life. Intrapartum antibiotic prophylaxis (IAP) for women at risk of transmitting GBS to their newborns has been effective in reducing, but not eliminating, the young infant GBS disease burden in many high income countries. However, identification of women at risk and administration of IAP is very difficult in many low and middle income country (LMIC) settings, and is not possible for home deliveries. Immunization of pregnant women with a GBS vaccine represents an alternate pathway to protecting newborns from GBS disease, through the transplacental antibody transfer to the fetus in utero. This approach to prevent GBS disease in young infants is currently under development, and is approaching late stage clinical evaluation.

This manuscript includes a review of the natural history of the disease, global disease burden estimates, diagnosis and existing control options in different settings, the biological rationale for a vaccine including previous supportive studies, analysis of current candidates in development, possible correlates of protection and current status of immunogenicity assays. Future potential vaccine development pathways to licensure and use in LMICs, trial design and implementation options are discussed, with the objective to provide a basis for reflection, rather than recommendations.

## Introduction

*Streptococcus agalactiae* is also known as Lancefield’s group B *Streptococcus* (GBS), and is a Gram-positive diplococcus, originally known for causing bovine mastitis ^1^. GBS remains the leading cause of neonatal sepsis and meningitis, and is associated with significant mortality and morbidity, including long-term neurodevelopmental sequelae ^2^. Disease risk is the highest during the first 3 months of life ^3^, the primary target for GBS disease control efforts, but risk of invasive GBS disease increases again later in life, in particular among pregnant women and adults with underlying conditions or older age ^1^.

Neonatal infections (sepsis and pneumonia) contribute importantly to deaths among children under 5 years of age globally, with the highest rates in low income countries, followed by middle income countries ^4^. The etiologies of neonatal infections in low income countries are poorly characterized but GBS likely contributes to this burden. A recent systematic review showed that neonatal GBS disease incidence and case fatality rates are highest among countries in sub-Saharan Africa. However, published data from this region remain sparse and the estimated numbers are still considered underestimates ^3^. In high-income countries, GBS emerged as a leading cause of neonatal infection in the 1970s for reasons that remain poorly understood. Many resource rich settings have experienced significant reductions in the incidence of early-onset disease (onset of disease during days 0–6 of life) after introduction of targeted administration of intrapartum intravenous antibiotics to women at risk of transmitting GBS to their newborns ^5, 6^. However, this intrapartum prophylaxis has not proven to be effective in preventing late-onset disease (disease onset during days 7–89 of life), and is not implemented in most high disease burden low-and middle-income countries (LMIC). Therefore, there has been a longstanding interest in developing a maternal vaccine against GBS to prevent disease in infants of vaccinated mothers.

Among various vaccine candidates, the glycoconjugate vaccines targeting GBS capsular polysaccharide (CPS) have been most studied, although common protein vaccines hold the appeal of broader coverage against circulating disease-causing strains. GBS vaccine development underwent an active phase in the 1990s. Although pre-clinical and early clinical studies showed promise, efforts slowed for a period, for a variety of reasons, including the strong success of intrapartum prophylaxis in reducing the early-onset disease burden in high income countries, and concerns about the acceptance and the liability coverages for maternal immunization. Recent years have experienced a wave of new activity in GBS vaccine development. Successes in rolling out pneumococcal conjugate, rotavirus, and *Haemophilus influenzae* type b vaccines to the world’s poorest countries through the GAVI alliance paved the way for future LMIC vaccine introductions. Finally there is a renewed interest in invigorating the maternal immunization platform, and several licensed products such as tetanus, influenza and pertussis vaccines are recommended for use among pregnant women in LMIC.

This review provides necessary background for non-GBS subject matter experts on issues of relevance to accelerating development of a GBS vaccine for LMIC. It draws almost exclusively on published literature or public information but alludes to some key activities of relevance that are anticipating publications in the near future. First we provide an overview of GBS disease and the global burden with a focus on GBS disease in infants (days 0–90 days), the primary prevention target for a maternal immunization program. This is followed by a summary of GBS diagnostics, and a review of intrapartum antibiotic prophylaxis (IAP), standards of care, strategies and impact. The next three sections provide relevant background in GBS vaccine development including a brief review of GBS virulence factors, the history of GBS vaccine development, and a review of safety and immunogenicity of current vaccine candidates, primarily from phase II studies of a trivalent glycoconjugate vaccine formulation. This section also reviews issues related to measuring serologic endpoints and the current status of establishing immune correlates of protection. The final three sections address cost-effectiveness analysis and other potential contributions of mathematical modeling to GBS vaccine decision-making; options regarding the planning and conduct of a phase III efficacy study; and different possible vaccine development pathways are presented. We conclude with a high level summary of key gaps in knowledge.

## Diseases and sequelae caused by GBS and population at risk

Given the purpose of this document, information used in the next two sections (“ Diseases and sequelae caused by GBS and population at risk” and “ GBS disease burden and serotype distribution”) is primarily from LMIC, supplemented with data from high-income countries whenever information from LMIC was not available.

### Early-onset neonatal disease

***Definition.*** Although definitions for early-onset neonatal disease vary, the most common include onset of GBS disease within 72 hours of birth or days 0–6 of life ^7^. See section on ‘ Considerations for licensure based on immune markers’ for candidate definitions for a phase III trial.

***Transmission.*** Early-onset disease is caused by vertical transmission through colonized mothers during or just before birth ^8^. GBS can ascend from the vagina to the amniotic fluid after onset of labor or rupture of membranes ^9^, although intrauterine infection without evidence of ruptured membranes has been reported ^10, 11^. GBS in the amniotic fluid can colonize the fetal skin or mucus membranes or can be aspirated into the fetal lungs, leading to an invasive infection ^12, 13^. Infants can also be exposed to GBS during passage through the birth canal and can become colonized at mucus membrane sites in the gastrointestinal or respiratory tracts. It has been estimated that in the absence of any intervention, approximately 50% of babies born to colonized mothers become colonized and 1–2% of them progress to develop invasive disease ^14– 16^.

***Risk factors.*** Risk factors for early-onset disease have been well described in resource-rich settings. A review of risk factors as established in United States studies showed that the strongest risk factor for neonatal disease was a positive maternal vaginal culture at delivery (Odds Ratio [OR]: 204) ^17^. Other risk factors include prolonged rupture of membranes, preterm delivery, GBS bacteriuria during pregnancy, birth of a previous infant with invasive GBS disease, maternal chorioamnionitis as evidenced by intrapartum fever, young maternal age, and low levels of antibody to type-specific capsular polysaccharide antigens ^18– 21^. Although few risk factor analyses have been conducted in LMIC, epidemiologic characteristics of case series from these settings ^22– 25^, as well as a risk factor analysis of early-onset neonatal sepsis in South Africa ^26^, suggest that the same risk factors play an important role in LMIC. Additionally, human immunodeficiency virus (HIV) infection in mothers has been shown to increase the risk of neonatal GBS disease. Recent studies from South Africa reported that HIV-infected women have lower GBS antibody concentrations and reduced transplacental antibody transfer compared to HIV-uninfected women ^27, 28^, and infants born to HIV infected mothers had lower anti-GBS surface binding antibody levels ^28^. However, maternal HIV infection appears to be more of a risk for late-onset disease compared to early-onset disease.

***Disease onset and clinical presentation.*** Most cases occur within the first 24 to 48 hours ^23, 25, 29– 31^, and more than half of early-onset disease occurs in term (≥37 weeks gestation) infants: studies from sub-Saharan Africa have reported the proportion of pre-term infants among infants with early-onset disease to range from 10 to 46% ^22, 24, 25^. Respiratory distress has been described as one of the most common presenting symptoms, ranging from 68% (South Africa, single hospital retrospective review, 1997–1999) ^25^ to 83% (South Africa, surveillance at three secondary-tertiary care hospitals, 2012–2014) ^24^. Early-onset disease most frequently manifests with bacteremia, and less frequently with meningitis. A study in Malawi demonstrated that about half (52%) of children with early-onset disease presented with sepsis (defined as GBS isolated from blood with no clinical evidence of pneumonia), 17% with meningitis, and 14% with probable meningitis (GBS isolated from blood and cerebrospinal fluid [CSF] findings consistent with meningitis) ^22^. In the US, infants with early-onset GBS disease present primarily with bacteremia (80%), meningitis (6%), or pneumonia (7%) ^32^. A recent study from Uganda suggests that neonatal GBS infection may be an under-recognized cause of cerebral hypoxic encephalopathy ^33^, although more data are needed to confirm the nature of the link between the two.

***Disease outcomes.*** Case fatality ratios reported in hospital-based studies from sub-Saharan Africa have ranged from 20% to 38% ^22, 24, 25^, with higher case fatality among preterm infants ^24^. A multi-country observational study conducted in Panama, Dominican Republic, and Hong Kong showed that the average number of days hospitalized ranged from 6 (Dominican Republic) to 15 (Hong Kong), with case fatality ratios ranging from 10% (Hong Kong) to 33% (Dominican Republic) ^34^. Case fatality ratios in resource rich settings are notably lower ( *e.g*., United States: 4–6%), yet a study from the United States reported a nearly eight times higher risk of death among preterm cases compared to term cases ^35^.

### Late-onset neonatal disease

***Definition.*** Late-onset infections occur among infants aged 7–89 days of life ^9, 36^. In some instances the period from day 3–89 is considered ^7^.

***Transmission.*** As with early-onset disease, development of late-onset GBS disease first requires adhesion of GBS to mucosal surfaces, followed by invasion across epithelial cells to gain entry to the bloodstream. Vertical transmission from colonized mothers can result in late-onset disease, although it is considered to play a less important role compared to early-onset disease ^37^, and IAP has not impacted the late-onset disease burden in countries that provide IAP ^38^. Nosocomial transmission, horizontal transmission from mother to infant after the perinatal period, and transmission from breast milk have also been described ^39– 42^, although it is unclear whether these are common routes of transmission ^38^.

***Risk factors.*** Risk factors for late-onset disease are less understood than those for early-onset disease, and prevention strategies for late-onset infections have not yet been identified. Some of the identified risk factors are similar to those of early-onset disease, such as preterm delivery and maternal GBS colonization ^43, 44^. More recent studies have shown that preterm delivery may be a major factor for late-onset disease, with each week of decreasing gestation associated with an increased risk of late-onset disease ^44, 45^. Another prospective cohort study from Italy also showed that preterm infants had an increased risk for late-onset disease ^46^.

As mentioned above, HIV exposure may be a greater risk for development of late-onset disease compared to early-onset disease: one study from South Africa reported that the risk ratio of the incidence of GBS disease was 1.7 (95% CI: 1.3–2.2) compared to HIV-unexposed infants for early-onset disease vs. 3.2 (95% CI: 2.3–4.4) for late-onset disease ^47^. Another South African study reported that the incidence of early-onset disease was similar between HIV-exposed and un-exposed (1.1 vs. 1.5; p=0.5) but there was a 4.7-fold greater risk (95% CI: 2.3 vs. 0.5; p<0.001) for late-onset disease ^24^. Similar results were reported from a study conducted in Belgium ^48^.

***Disease onset and clinical presentation.*** Studies reported different proportions of preterm infants (<37 weeks) among late-onset cases: 49% in the United States ^38^, 25% in South Africa ^24^, and 14% in Malawi ^22^, suggesting this proportion may be lower in LMIC than in high-income countries. A study from Italy showed that term infants develop disease earlier (median 23 days, interquartile range [IQR] 15–42) compared to preterm infants (median 39 days, IQR 28–58) ^46^.

The proportion of infants with late-onset disease presenting with meningitis is higher compared to infants with early-onset disease, and data from sub-Saharan Africa reported that meningitis is one of the leading clinical presentations for late-onset disease (33–59%) ^22, 24, 25^. Data from the United States show that about 26% of infants with late-onset disease presented with meningitis, while 67% had bacteremia without a focus of infection ^38^.

***Disease outcomes.*** Because of the higher proportion of meningitis cases among infants with late-onset disease, risk of long-term neurologic sequelae may be higher among survivors of late-onset disease compared with infants surviving early-onset disease ^49^. A study from South Africa showed that GBS-affected infants were >13 times more likely to have neurological sequelae at 6 months of age compared to controls, defined as abnormal Denver-II assessments (in the following domains: gross motor, fine motor, language and personal/social) or presence of hypertonia or hyper-reflexia ^24^. Results from the United Kingdom showed that 22% of survivors of neonatal meningitis (≤28 days of life) had mild to moderate sequelae ( *e.g*., isolated hydrocephalus, isolated epilepsy, mild learning problems, mild cerebral palsy), and 14% had severe sequelae ( *e.g*., cerebral palsy, global delay, significant learning problems) at 9–10 years of age ^50^. Another multi-center study from the United States described similar percentages of neurologic sequelae among GBS meningitis survivors: 25% with mild-to moderate impairment, and 19% with severe impairment at a mean age of 7 (range 3–12) years ^2^.

Reported case-fatality ratios are lower compared to those of early-onset disease ^3, 22, 24, 25, 32^. In the systematic review by Edmond *et al*., the pooled result of all studies reporting case fatality for early-onset disease was 12.1% (95% CI 6.2–18.3) and was 6.8% (95% CI 10.8–14.9) for late-onset disease. A more recent systematic review conducted by Sinha *et al*. reported that health facility-based studies from Malawi and South Africa reported case fatality ratios ranging from 20–38% for early-onset disease and 14–29% for late-onset disease (meta-analysis was not done due to heterogeneity in numerator and denominator) ^51^.

### Other perinatal complications (preterm delivery, stillbirth)

***Preterm delivery.*** GBS colonization during pregnancy has been associated with preterm delivery ^52, 53^, although the association is less clear than the association between colonization and early-onset disease. A systematic review which included 20 studies from 10 different countries summarized results by study design: results from cross-sectional studies conducted at the time of delivery had a pooled OR of 1.75 (95% CI 1.43–2.14) for preterm delivery between GBS colonized mothers and non-colonized mothers, and 1.59 (95% CI 1.03–2.44) for case-control studies that matched mothers with preterm delivery with mothers with the same gestational age, but not in labor. Whether colonization causes preterm delivery is still a matter of debate. A systematic review of cohort studies evaluating the odds of preterm delivery according to colonization status during pregnancy were inconclusive (pooled OR: 1.06; 95% CI 0.95–1.19) ^54^.

***Stillbirth.*** GBS has also been associated with spontaneous abortions and stillbirths. A retrospective study conducted in Australia which reviewed causes of spontaneous abortions (between 16 to 26 weeks gestation) among those with autopsy and microbiological cultures available showed that GBS was the most significant pathogen, often being the sole pathogen recovered, and found both in babies born to women with intact as well as ruptured membranes ^55^. A study using United States population-based surveillance data showed that 24% of invasive GBS infections during pregnancy resulted in septic abortions and/or stillbirths, a higher proportion than observed for pregnancy-associated invasive infections with *Streptococcus pneumoniae* (8%) or group A *Streptococcus* (6%) ^56^. Invasive GBS infections are however infrequent among pregnant women, whereas GBS colonization is much more common. Estimating the burden of GBS-related stillbirths is challenging, even in high income countries.

### Maternal pregnancy-associated and postpartum GBS disease

GBS can cause urinary tract infection, chorioamnionitis, endometritis, and bacteremia in women ^49^. Women during pregnancy and shortly after are at a higher risk of developing invasive GBS disease compared to non-pregnant women of the same age group ^56^. Data on pregnancy- and postpartum-associated GBS disease are limited, even in resource-rich settings, and we are not aware of data from LMIC. Data from United States population-based surveillance showed that GBS bacteremia without focus was the most common presentation both during pregnancy (43%) and the postpartum period (32%), followed by chorioamnionitis (33%) in pregnant women, and endometritis (25%) in postpartum ^56^. Pneumonia and puerperal sepsis have also been reported ^35^. Unlike influenza, invasive GBS infection during pregnancy or the postpartum period was not associated with a longer hospital stay, an indicator of disease severity, or increased mortality risk, compared to non-pregnant women ^56^. Most (81%) of these pregnancy-associated invasive infections occurred in absence of additional underlying conditions ^35^.

Vaginal GBS colonization is considered to be a risk for maternal chorioamnionitis and postpartum endometritis ^57, 58^. Some studies have suggested that GBS bacteriuria during pregnancy, possibly an indicator for heavy colonization ^59, 60^, may be associated with an increased risk for adverse obstetric outcomes, such as habitual abortion, intrauterine growth restriction, preterm labor, chorioamnionitis and premature rupture of membranes ^14, 61^. However, other studies have shown that asymptomatic GBS bacteriuria during pregnancy correlates poorly with GBS genital cultures at 35–37 weeks ^62^ or at delivery ^63^. Both GBS vaginal colonization and bacteriuria are commonly asymptomatic among pregnant women.

### Disease in non-pregnant adults

An increasing incidence of invasive GBS disease has been reported among non-pregnant adults in recent years, primarily from high-income countries where surveillance for invasive GBS disease among all ages has been conducted ^35, 64– 67^. Results from population surveillance from the United States showed a doubling of invasive GBS cases among non-pregnant adults (≥18 years) between 1990 and 2007 ^67^. The only multi-province surveillance for invasive GBS disease among non-pregnant adults in LMIC that we are aware of comes from Thailand, where GBS was the leading pathogen in an invasive bacterial diseases surveillance system ^68^.

Skin- and soft- tissue infections are one of the most frequent clinical presentations in adults, although clinical syndromes associated with invasive GBS infections are variable, including bacteremia, pneumonia, bone and joint infections, urosepsis, endocarditis, meningitis, and intravenous catheter infections ^69^. A study from South Africa showed that soft tissue abscesses and pneumonia accounted for 70% of the presentations and reported an overall 35% mortality among all identified GBS cases ^70^. Another study from Malaysia showed that skin and soft-tissue infections accounted for >70% of all GBS infections ^71^. The majority of disease occurs in people with significant underlying conditions, particularly diabetes mellitus. The proportion of subjects with diabetes reported in population-based surveillance for invasive disease ranged from 20% in Canada ^72^ to ≥40% in the United States of non-pregnant adults aged ≥15 years ^35, 67^. Estimates of the prevalence of diabetes among adults with invasive GBS infections from other countries come primarily from single institution studies, and vary from 28% in Soweto, South Africa to 71% in Malaysia ^64, 70, 71, 73, 74^. Other conditions associated with increased risk of invasive GBS disease among non-pregnant adults include atherosclerotic cardiovascular disease, obesity, cancer, heart failure, and renal disease ^67^. Age is also a risk factor; data from the United States, Europe and Thailand have shown that incidence rates for invasive GBS are highest among adults aged ≥65 years ^65– 68^.

## GBS disease burden and serotype distribution

### Challenges in estimating global disease burden

Quantifying the burden of neonatal GBS disease remains a challenge even in high-income countries: clinical characteristics are non-specific and often difficult to differentiate from non-infectious causes ^75^. Invasive infections are most commonly diagnosed based on isolation of GBS from a normally sterile site ( *e.g*., blood, cerebrospinal fluid) in microbiological culture; however, sensitivity of blood culture varies depending on the bacterial load, blood collection, and culture method, and typically requires 36 to 48 hours for positive results to become available ^75^. Estimating GBS disease burden in LMIC is even more difficult: a portion of births may occur outside of hospital settings; facility-born infants may be discharged quickly after birth; care seeking, particularly early in life, may be limited; access to care, particularly in rural areas may pose challenges; and health facilities may lack access to diagnostic tests or laboratory capacity or resources to diagnose GBS infection. As a result, particularly for early-onset disease, most of which occurs within the first 24–48 hours of life, GBS disease is likely underrepresented in studies from these settings ^76^. Finally, incidence of neonatal GBS varies regionally ^3, 14^. IAP use should be considered in making regional comparisons, as IAP, an intervention known to reduce the risk of early-onset disease, is widespread in many resource-rich settings but rarely implemented in LMIC ^3^.

As a result of these challenges, and the relative paucity of invasive disease data from LMIC, some researchers have focused on GBS colonization as a surrogate measure for neonatal disease. However, different studies in resource rich settings have reported similar and high maternal colonization prevalence but different neonatal disease incidence ^3, 14^, suggesting that the relationship between maternal colonization and newborn disease is not simple.

Estimating the invasive GBS disease burden in pregnant women is difficult due to the paucity of data from LMIC, and the common clinical practice of empiric treatment in absence of a definitive diagnosis for postpartum infections. Estimating the burden of GBS-related stillbirths poses challenges even in high-income countries. First, there is inconsistency in the definition used for stillbirth. For international comparison, the World Health Organization (WHO) defines stillbirth as a baby born with no signs of life at or after 28 weeks gestation ^77^. However, various definitions of stillbirth have been used, making comparisons difficult between countries, or even within the same country ^78– 80^. In addition, stillbirths are not reported in national surveillance systems in about 90 countries ^80, 81^, and even where stillbirth is included in vital reporting systems, the causes of stillbirth are generally not recorded ^82^. Diagnostic procedures may not be conducted or may not be available, and even if performed, there may be difficulties in producing valid results, as pathologic changes could have occurred before the time of investigation. Because GBS is a common colonizing organism of the birth canal, distinguishing the presence of GBS (for example in amniotic fluid or placenta or even fetal tissue) due to colonization from a direct role of GBS in fetal death adds further challenge in identifying the cause of death ^82^.

### Disease burden and serotype distribution by WHO region

***Infants aged <90 days.*** Neonatal sepsis (in infants aged <1 month) is one of the leading causes of neonatal deaths globally ^83^, and among the WHO regions, the burden is the highest in the African region, where it was estimated to cause 5.3 deaths per 1,000 live births in 2012 ^84^.

A group at the London School of Hygiene and Tropical Medicine is updating the global GBS disease burden estimates, focusing on neonatal and maternal disease; results are expected in 2017. The most recent systematic review and meta-analysis currently available of invasive GBS disease in infants aged <90 days was published in 2012. This review showed that incidence of GBS disease was the highest in Africa (1.21 per 1,000 live births, 95% CI 0.50–1.91), followed by the Americas (0.67 per 1,000 births, 95% CI 0.54–0.80), and lowest in southeast Asia (0.02 per 1,000 live births, 95% Ci -0.03 to 0.07) ^3^ ( Table 1). While IAP is common in the Americas it is rarely used in Africa or Southeast Asia. Incidence of early-onset disease and late-onset disease was also the highest in Africa (0.53 and 0.24 per 1,000 live births, respectively). However, only four studies were available for incidence estimates in Africa (Kenya, Malawi, Nigeria, South Africa). A more recent systematic review based on additional studies from sub-Saharan Africa reported a somewhat higher estimated incidence of neonatal disease: 1.3 cases per 1,000 births for early-onset disease (Kenya, Malawi, Mozambique, Nigeria, South Africa, Zimbabwe) and 0.73 per 1,000 births for late-onset disease (Kenya, Malawi, Mozambique, Nigeria, South Africa, Zimbabwe) ^51^, although the authors believe this is still an underestimation of the actual incidence given the challenges in collecting data in these countries. A recent study of early-onset sepsis in Soweto, South Africa that used both blood culture and a real-time polymerase chain reaction test for GBS on whole blood estimated an incidence of early-onset GBS disease of 1.8 per 1000 live births, higher than the estimate of 1.3 per 1000 live births based on blood culture detections alone (Sithembiso Velaphi, SANISA study, in preparation). This difference underscores that invasive disease estimates from blood culture are minimum estimates unless they take into account blood culture sensitivity.

**Table 1.** Estimated GBS disease incidence among infants with disease onset 0–89 days, by region (adapted from 3).

Region (year of publication of included studies)	Countries included in review (number of studies)	Pooled estimate of incidence per 1,000 live births (95% CI)
Europe (2001–2011)	Czech Republic (1), Denmark (2), France (1), Germany (1), Italy (1), Netherlands (2), Norway (2), Portugal (1), Slovakia (1), Spain (3), Sweden (1), UK (4)	0.57 (0.44–0.71)
The Americas (2002–2009)	Antigua and Barbuda (1), Brazil (1), Jamaica (2), USA (12)	0.67 (0.54–0.80)
Eastern Mediterranean (2002–2009)	Iraq (1), Kuwait (1), Saudi Arabia (1), Tunisia (1)	0.35 (0.07–0.62)
Western Pacific (2004–2009)	Australia (1), Australia and New Zealand (2), Macau (1), Malaysia (1), South Korea (1), Singapore (1)	0.15 (0.04–0.27)
Southeast Asia (2002–2009)	Bangladesh (1), India (2), Thailand (2)	0.02 (-0.03–0.07)
Africa (2005–2009)	Kenya (1), Malawi (1), Nigeria (1), South Africa (1)	1.21 (0.50–1.91)

The low reported incidence of neonatal GBS disease from South Asia poses a puzzle: is this an accurate reflection of the disease burden, or is it a reflection of under-ascertainment due to the challenges of capturing specimens from ill newborns, particularly on day 0 of life, in this region? The Aetiology of Newborn Infections in South Asia (ANISA) ^85^ study attempted to fill this gap by conducting population-based surveillance and etiologic evaluation of possible serious infections among newborns <60 days of age in the community in catchment areas in Bangladesh, Pakistan and India ^86^. The study faced some anticipated challenges in registering babies on day 0 of life (88% of all live births were enrolled in the surveillance and 74% of those enrolled were visited within 24 hours) and in capturing specimens from babies who died, particularly on day 0 of life. The study nevertheless included samples from over 970 babies with possible serious infection onset on day 0 of life. At all study sites, presence of GBS was assessed by blood culture, and by PCR on whole blood and PCR on nasopharyngeal/oropharyngeal samples on both ill babies and healthy controls. Colonization was detected, providing evidence that GBS is present in this region, consistent with other studies in Bangladesh and India ^87^. However, culture-confirmed infections were rare among babies with possible serious infection in the ANISA study; culture-confirmed GBS infections were identified at the Sylhet site (Bangladesh) and Vellore site (India). Fuller results are under preparation for publication.

**Figure 1.**
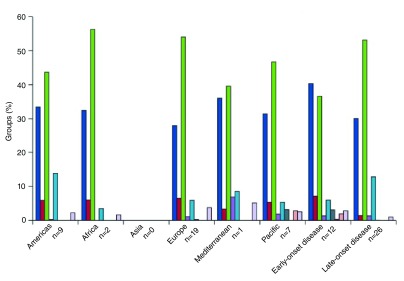
Global distribution of GBS serotypes by WHO region and disease onset, 1980–2011 (N=38 papers reviewed in total) ^3^. This figure has been reproduced with permission from Elsevier ^3^. n indicates the number of articles included in the review for each region and disease category.

Edmond and colleagues reviewed available data on global serotype distribution. Serotype III accounted for almost half of all isolates, followed by serotypes Ia, Ib, II, and V, and, this trend was similar across all WHO regions. Five serotypes (Ia, Ib, II, III, V) accounted for more than 85% of serotypes in all regions with available data: 98% in Africa, 96% in the Americas, 93% in Europe, 89% in western Pacific, and 88% in the eastern Mediterranean ^3^ ( Figure 1). However, serotype studies from low-income countries and Southeast Asia were not identified in this review ( Table 2). Although not limited to neonates, a recent report from Vaccine Preventable Infections Surveillance conducted in Thailand sheds some light on serotype distribution of GBS disease in the southeast Asian region: among children aged <5 years with invasive GBS disease, serotype III was the most frequently isolated (approximately 50%), followed by Ia and Ib (approximately 13% each) ^88^. In Edmond’s global review, the proportion of serotype III isolates was larger for late-onset compared to early-onset disease (53% vs. 37%) ^3^ ( Figure 1). A more recent review from sub-Saharan Africa showed similar results: the five serotypes (Ia, Ib, II, III and V) accounted for 97% of early-onset disease and 98% of late-onset disease, and the proportion of serotype III was higher in late-onset cases (79%) than in early-onset cases (54%) ^51^.

**Table 2.** Summary of countries with serotype data available from neonatal GBS disease and serotype distribution, by WHO region (adapted from 3).

WHO region	Number of serotype articles available out of a total of 38 (1980–2011)	Countries reporting serotype
Americas	9 (24%)	U.S. (6), Canada (2), Argentina (1)
Africa	2 (5%)	South Africa (1), Nigeria (1)
Southeast Asia	0	NA
Europe	19 (50%)	Czech Republic (1), Denmark (3), France (2), Sweden (2), U.K. (2), Portugal (2), Germany (2), Netherlands (1), Israel (1), Romania (1), Finland (2)
Eastern Mediterranean	1 (3%)	Morocco (1)
Western Pacific	7 (18%)	Japan (5), Australia (1), Singapore (1)

***Maternal colonization.*** Ascertainment of maternal genital GBS colonization varies according to the specimens collected ( *e.g.*, vaginal sampling only vs. rectovaginal sampling), the culture medium, specimen transport and processing procedures and timing. In general, rectovaginal sampling has a higher yield than vaginal sampling only ^89, 90^, and use of selective broth media is better compared to nonselective blood agar ^89^. A review estimated the prevalence of genital colonization in pregnant women to be around 13% globally, although included studies used various laboratory methods ^91^. When restricted to studies which were considered to have used adequate methods (collection site including the vagina and using selective broth media), the estimated overall prevalence was 18% with regional variation: 12% in India/Pakistan, 19% in Asia/Pacific, 19% in sub-Saharan Africa, 22% in Middle East/North Africa, and 14% in the Americas (no data from Europe included) ^91^. Regional variation was also reported in a multi-country cross-sectional study among pregnant women between 20 and 32 weeks gestation which used a standardized laboratory method (specimens collected from the cervix, lower vaginal wall, and urine and used selective enrichment broth) and showed that overall colonization prevalence was 11%, ranging from 8% in Manila, the Philippines, to 22% in Philadelphia, United States, which may reflect geographic differences in disease burden ^92^.

GBS colonization is known to fluctuate during pregnancy, and a recent longitudinal study in South Africa reported that acquisition rates and the duration of colonization differ according to GBS serotype ^93^. Serotype distribution in colonized mothers may not correlate directly with serotypes causing invasive neonatal disease, as invasiveness appear to be different according to GBS serotype ^94^. However, colonization data may provide some insight into circulating GBS serotypes in regions where data from invasive disease are limited, especially Southeast Asia. A recent systematic review from sub-Saharan Africa showed that serotype III (>30%), Ia and V (both >20%) were the most frequently isolated ^51^. Another study that took place on the Thai-Myanmar border showed that serotype II was the most frequently isolated serotype (24%), followed by Ia, VI, III, and V ^95^. Results from a multi-country study showed that overall, serotype III (17.8%) was the most frequently isolated serotype, followed by serotypes V (17%) and Ia (5%); however, serotype III was not isolated in two of the sites (Philadelphia, United States and Yangon, Myanmar), whereas serotype VII was the most frequently isolated serotype in Khon Kaen, Thailand, and was the only site that reported this serotype ^92^. The GBS global serotype distribution appears more diverse than previously reported; a recent review of maternal GBS colonization showed significant heterogeneity across and within regions ^96^. Additionally the modeling team led by the London School of Hygiene and Tropical Medicine is including a comprehensive review of maternal GBS colonization, risk of neonatal disease, neonatal disease incidence and impairment outcomes as part of their update of GBS disease burden estimates. They will also review data on GBS-related stillbirth, GBS-related preterm birth and review data on the association of GBS disease with neonatal encephalopathy.

***Pregnancy-associated GBS disease and stillbirths.*** As described above, few data are available on the incidence of invasive GBS disease among pregnant and postpartum women in low- and middle-income countries. A study from the United States showed that the incidence of invasive GBS disease was 0.04 (95% CI 0.03–0.05) per 1000 women-years for pregnant women, 0.49 (95% CI 0.36–0.64) per 1000 women-years for postpartum women, and 0.02 (95% CI 0.02–0.02) for non-pregnant women between the ages 15–44 years ^56^.

A recent systematic review evaluated the incidence of GBS-related stillbirth (defined as at ≥20 weeks gestation most likely caused by GBS infection, as confirmed by a GBS-positive culture sample from the placenta and/or amniotic fluid and/or a normally sterile site) ^97^. GBS-related stillbirth rates ranged from 0.04–0.9 per 1,000 births with highest reported from a small study in the United States ^11^, and the proportion of stillbirths attributable to GBS infection ranged from 0–12% ^97^; however, the review was limited by inconsistencies in stillbirth definitions and diagnostic methods and the number of studies available that met the inclusion criteria, particularly those from low- and middle-income countries to assess the burden of GBS-related stillbirth worldwide. Stillbirth data from Kenya were recently published ^98^, and data from South Africa are currently being evaluated and are expected to be available, soon. These were both prospective studies that attempted to capture meaningful specimens from stillbirths for diagnostics and that applied similar, although not identical case definitions for a GBS-related stillbirth. In South Africa, preliminary estimates suggest GBS-related stillbirth incidence may be similar to that of early-onset GBS disease incidence (personal communication, Dr. Shabir Madhi).

***Disease in non-pregnant adults.*** Annual incidence rates have been reported primarily from North America and Europe, ranging from 1.5 per 100,000 population (Spain, ages 21–100 years, 1992–1999) ^73^ to 7.3 per 100,000 population (United States, ages 18–105 years, 2007) ^67^, and the rates tend to be higher with increasing age ^35, 66, 67^. A population-based surveillance of invasive bacterial infections conducted in Thailand between 2010 and 2013 showed that the average annual incidence of invasive GBS disease is the highest among those aged ≥70 years (23 per 100,000 population ^68^, similar to incidence reported among adults aged ≥65 years in 2005 in the United States (25.3 per 100,000 population) ^35^. As seen when evaluating GBS disease incidence in other age groups or GBS colonization, geographic variation has been noted in the serotype distribution: reports from North America shows that serotype V is the most frequently isolated serotype in adult GBS disease, representing approximately 30% of the isolates as opposed to 11% for serotype III ^67, 72^, whereas reports from Europe show that serotype V (approximately 20%) was less frequent compared to serotype III (25–30%) ^66, 99^. A surveillance report from Thailand shows that among adults aged 21 years or older, serotypes Ia, II, II, V and VI represented >90% of cases, with serotype III being the most common (approximately 48%) ^88^.

## Diagnosis and treatment of GBS disease

### Diagnosis

This section summarizes clinical and laboratory methods commonly used for diagnosing GBS disease. For summary of case definitions used in published studies and discussion on candidate case definitions for phase III studies.

***Newborns/Young infants***

**Clinical evaluation of sick children** Integrated Management of Childhood Illness (IMCI) was developed jointly by WHO and the United Nations International Children’s Fund (UNICEF) to promote the accurate identification and appropriate treatment of common childhood illnesses at first-level health facilities in low-income countries, where health workers rely on patients’ history, and signs and symptoms to determine a course of management. Infants <2 months of age are assessed for signs of very severe disease: not feeding well, convulsions, fast breathing [≥60 breaths/min], severe chest indrawing, fever [≥37.5°C], low body temperature [<35.5°C], movement only when stimulated or no movement at all. These clinical syndromes which warrant urgent referral of young infants to hospitals are defined as possible serious bacterial infection (PSBI) ^100^. Rates of PSBI among newborns young infants in LMIC can be very high (80 cases/1000 live births or higher) ^101^. Even in the higher middle income country setting of South Africa, hospital admission for physician-suspected early-onset sepsis occurred at a rate of approximately 30 cases/1000 live births ^15^. WHO guidance recommends that newborns presenting with signs of PSBI should be admitted to the hospital and blood cultures and lumber punctures should be obtained whenever possible before starting antibiotics ^102^. Additionally, the United States Centers for Disease Control and Prevention (CDC) GBS prevention guidelines recommend a blood culture at birth for infants born to mothers with chorioamnionitis, even if the infant is well-appearing ^9^.

IMCI has a separate set of algorithms for children 2 through 59 months. Children are first assessed for general danger signs (unable to drink or breastfeed, child vomits everything, lethargic or unconscious, had convulsions or actively convulsing). If stiff neck or general danger signs are present in a child with fever, administration of antibiotics and urgent referral are recommended ^103^.

**Laboratory detection of invasive disease** Confirmation of invasive GBS disease requires isolation of GBS from a normally sterile site ( *e.g*., blood, CSF), which is usually done by collecting cultures. Automated blood culturing systems have improved the practice of blood culture: the automated system automatically detects microbial growth by monitoring microbial CO _2_ detection, and eliminates the need for manual inspection or examination. In addition, growth of aerobes and facultative anaerobes are promoted by agitating culture bottles ^104^. However, a recent review reported that many studies from LMIC used manual culture methods, with lower GBS incidence rates compared to studies using automated culture methods ^105^. Therefore, differences in the culture methodology used can result in variation in reported GBS disease burden.

Rates of culture-confirmed infection are typically an order of magnitude or more lower than rates of clinical sepsis, although the culture positivity rate varies according to the criteria for collecting culture and how samples were collected. It is known that the likelihood of pathogen isolation increases with the quantity of blood submitted for culture, and for neonates, at least 0.5 to 1 ml of blood is recommended ^7, 106^. Because of the small blood volumes that can be obtained from newborns and young infants it is also important to use pediatric rather than adult blood culture bottles. In real use many cultures contain inadequate amounts of blood ^107, 108^. The yield of blood culture also varies with organism density in the blood. A study in infants 0–2 months of age showed that about half of the cultures positive for GBS had a very low organism density (≤1.0 cfu/ml) ^109^. Based on an estimate from a study comparing the yield of pathogens from blood culture using blood samples with various volumes and bacterial load, the sensitivity of blood culture to detect low-level GBS bacteremia (1.0 cfu/ml) could vary from 44% (0.5 ml collected) to 98% (2 ml) ^110^.

CSF analysis by lumbar puncture is the gold standard to diagnose meningitis. It is considered that up to 23% of neonates with bacteremia will also have concomitant meningitis, and that up to 38% of those with meningitis will have a negative blood culture ^7^. Therefore, children suspected of meningitis should undergo lumbar puncture to assess the CSF whenever possible. Laboratory methods to identify GBS are summarized in Table 3. Cloudy CSF, elevated CSF leukocyte counts, low CSF glucose (e.g., < 1.5 mmol/litre or a ratio of CSF to serum glucose of ≤0.4), elevated CSF protein ( *e.g*., > 0.4 g/litre), and positive Gram stain results indicate presence of meningitis, and treatment should be started immediately while awaiting culture results ^102^.

**Table 3.** Laboratory methods for identification of GBS from specific sample types.

Sample type	Accepted laboratory methods
Bacterial isolate	Streptococcal grouping latex agglutination tests; DNA probe test (e.g., AccuProbe); nucleic acid amplification test; CAMP and hippurate tests (for presumptive identification); Chromogenic agars specific for GBS identification; MALDI-TOF MS
Colonization swabs Enrichment broth Bacterial isolation	Pigmented broth, DNA probe, latex agglutination, NAAT See bacterial isolate section above
CSF	Bacterial culture and species identification (see bacterial isolate section above), latex agglutination test, NAAT
Blood	Bacterial culture and species identification as above (automated blood culture methods preferred): gold standard; NAAT on whole blood: can increase detection yield but a low, non-zero portion of healthy controls also test positive by this method

CAMP: Christie, Atkinson, Munch, Peterson; GBS: group B *Streptococcus*; MALDI-TOF MS: Matrix-Assisted Laser Desorption Ionization-Time of Flight Mass Spectrometry; NAAT: nucleic acid amplification test

Once bacterial isolates suggestive of GBS are identified, various laboratory methods including culture-based methods and high sensitivity latex agglutination tests can be used for GBS identification (see Table 3). More recently, nucleic acid amplification tests (NAAT) ( *e.g*., polymerase chain reaction) have allowed direct GBS identification from clinical samples. Some studies have used NAAT in addition to culture in order to improve the detection of cases.

**Neonatal colonization** It is estimated that about half of neonates exposed to GBS by their colonized mothers become colonized with GBS, and only a small proportion of those develop invasive disease. Neonatal GBS colonization results from exposure to and swallowing of GBS-infected amniotic fluid or maternal vaginal secretions. External auditory canal cultures are more likely to yield GBS in the first 24 hours of life compared to other sites, and isolation of organisms from the ear canal is a surrogate for the degree of contamination from amniotic fluid and vaginal secretions sequestered during the birth process. After the first 48 hours of life, throat and rectal sites are the best sources for detection of GBS, and positive cultures indicate true colonization ( *i.e*., multiplication of organisms at mucous membrane sites), not just maternal exposure ^111^.

***Pregnant and postpartum women***

**Maternal colonization** Maternal colonization can be assessed by collecting swabs from the vagina and the rectum from pregnant women ^9^. Swabs are inoculated into a selective broth medium, and subcultured on to an agar plate for bacterial isolation. If enriched selective broth media is used, GBS can be determined faster (see Table 3).

**Chorioamnionitis** The diagnosis and reporting of chorioamnionitis varies widely. Some consider histopathologic diagnosis as the gold standard ^112^. Amniotic fluid sampling and culture can be used in the diagnosis of chorioamnionitis, however, diagnosis of chorioamnionitis is often made clinically due to challenges in accessing uncontaminated amniotic fluid or placenta for culture ^113, 114^. Culture of the fluid may be conducted, but may have limited clinical utility due to the potential colonization of the amniotic fluid and the time it takes to obtain results ^114^. In addition, the infectious etiology is often polymicrobial ^115^. Fever in a pregnant women is the most important clinical sign of chorioamnionitis. Other key clinical findings associated with clinical chorioamnionitis include uterine fundal tenderness, maternal tachycardia (>100/min), fetal tachycardia (>160/min), and purulent or foul amniotic fluid ^113^. The WHO reference material lists fever (≥38.0°C) in pregnant women with foul-smelling watery discharge after 22 weeks and abdominal pain as symptoms typically present among pregnant women with chorioamnionitis ^116^.

***Endometritis.*** The diagnosis of endometritis is also often made clinically, and is often due to polymicrobial infection ^117^. Clinically, endometritis presents as fever, uterine tenderness, abdominal pain, and a purulent lochia or a positive culture of endometrial fluid or tissue ^118^. Positive blood cultures may help identify the bacterial etiology, as bacteremia may be present in up to 20% of women ^118^. In the WHO clinical guidance, fever in women after childbirth with lower abdominal pain, purulent, foul-smelling lochia and tender uterus is described as signs and symptoms typically present in women with endometritis ^116^.

***Stillbirths.*** Identifying infection as a cause of stillbirth is challenging: it is often difficult to determine the cause of stillbirth, and organism isolation on the placenta or the surface of the fetus does not prove causality ^119^. Pregnant women may be colonized with GBS, and could contaminate the fetus or the placenta after membrane rupture or vaginal contamination during delivery ^119^. In a recently published systematic review of 17 studies of GBS-related stillbirths, diagnosis was made based on a range of laboratory methods: culture confirmation from placenta (eight studies), blood/CSF (12 studies), amniotic fluid (two studies), and internal organs (eight studies) ^97^. Careful placental histologic examination and autopsy are considered to be more useful in identifying the cause of stillbirths ^119, 120^, and culture of fetal heart blood or fluid from uncontaminated fetal sites during autopsy may help identify the infectious cause ^119^.

### GBS typing methods

***Serological methods.*** Serological classification of GBS is based on the identification of capsular polysaccharides and protein antigens ^121^. Capsular polysaccharide is currently the most advanced glycoconjugate vaccine target, and currently ten serotypes have been described (Ia, Ib, II–IX). Several serological methods have been used for serotyping ( *e.g*., Lancefield capillary precipitin method, double immunodiffusion ^122^, coaggulutination ^123^, enzyme immunoassay ^124^, latex agglutination ^125^). The Lancefield capillary precipitin method is considered as the “gold standard” ^1, 122^. One of the most common methods for capsular polysaccharide serologic typing is the latex agglutination method, using antibodies specific for the 10 recognized capsular polysaccharides ^126^. In a recent report of a multicenter external quality assessment of molecular and serological typing conducted in 14 institutions in 13 European countries, the commercially available latex agglutination method was the most widely used typing method, with a typeability value (number of accurate results/total number of tests performed) of >90% ^121^. Limitations of serological methods include failure to type an isolate (~4–9% are classified as non-typeable) due to lack of or low expression of capsular polysaccharide under experimental conditions, the presence of reversible non-encapsulated variants, or, although rare, expression of a new capsular serotype ^1, 126^. In addition, results are dependent on the quality of the antibodies used and on the experience of the laboratory ^126^.

***Molecular typing methods***

**Serotyping** As an alternative to serological serotyping methods, molecular approaches based on the detection of capsular gene typing have been developed in recent years. Molecular methods include polymerase chain reaction (PCR) in conjunction with sequencing, hybridization, or enzymatic restriction cleavage pattern analysis, and multiplex-PCR approaches ^1, 126– 128^. These molecular approaches are attractive because they have made it possible to assign a molecular serotype to otherwise nontypeable isolates by serologic methods, and because they are reproducible, specific, easy to perform, and suited for capsular polysaccharide typing in large-scale epidemiological studies ^1^. However, PCR serotyping could potentially misclassify certain serotypes ^126^. Also, PCR serotyping does not reveal if the capsular polysaccharide gene locus detected is actually expressed as a polysaccharide capsule ^126^. Recently, Sheppard and colleagues have conducted whole genome sequencing to determine serotype with promising results ^129^. Although the method currently may not be cost-effective merely for determining serotype ^129^, the whole genome sequencing platform can be used to obtain genotyping data of the strains, as described below, as well as in depth analyses of strains within clonal complexes ^130^.

**Genotyping** Molecular typing methods have been used for further characterization of GBS and are useful in distinguishing different GBS strains in epidemiological studies ^121^. Examples of methods include restriction fragment length polymorphism (RFLP) ^131^, pulsed-field gel electrophoresis (PFGE) ^132^, multilocus sequence-typing (MLST) ^133^ and more recently DNA microarray-based typing ^134^. Whole genome sequencing has also enabled the investigation of large and small scale genetic changes in comprehensive collections of GBS strains, thereby permitting enhanced understanding of the diversity of the organism ^135^. See Box 1 for definitions of serotype, genotype, strain, and clonal complex.

Box 1. **Definitions of serotype, genotype, strain, and clonal complex ^1, 327^**

Serotype: type of antigenically variable polysaccharide capsule

Genotype: the genetic makeup of an organism or a group of organisms with reference to a single trait, set of traits, or an entire complex of traits

Strain: a single isolate of any bacterial population and any laboratory induced variants thereof

Clonal complex: a group of bacterial strains derived from a recent common ancestor that share many alleles at various phylogenetically informative loci. A clonal complex generally includes the ancestral genotype and strains with minor variation

### Treatment

***Newborns/young infants.*** IMCI recommends hospitalization and intramuscular or intravenous treatment of all infants meeting the case definition for PSBI ^136^. The recommended antibiotic selection for management of “serious bacterial infection” and “meningitis” in infants aged <2 months is ampicillin and gentamicin ( Table 4) ^102^. To date GBS remains universally susceptible to beta lactam antibiotics so penicillin and ampicillin remain effective therapeutic agents. The 2010 CDC guidelines recommend providing antibiotic therapy pending culture results for well-appearing newborns whose mothers had suspected chorioamnionitis ^9^. The WHO recommends providing prophylactic intramuscular (IM) or intravenous (IV) ampicillin and gentamicin in neonates with documented risk factors for infection (see Table 4) ^102^.

**Table 4.** Summary of recommended management of severe bacterial disease in young infants and perinatal infections in selected guidelines from the World Health Organization.

Prevention of neonatal infections
Give prophylactic antibiotics only to neonates with documented risk factors for infection ^102^: • Membranes ruptures >18 hours before delivery • Mother had fever >38°C before delivery or during labor • Amniotic fluid was foul-smelling or purulent Give IM or IV ampicillin and gentamicin for at least 2 days and reassess; continue treatment only if there are signs of sepsis (or a positive blood culture)
Management of infants aged <2 months
**Infants <2 months with any signs of very severe disease ^103^:** • Give first dose of intramuscular antibiotics (ampicillin 50mg/kg and gentamicin 5mg/kg [age <7 days] or 7.5mg/kg [age ≥7days]) • Treat to prevent low blood sugar • Refer URGENTLY to hospital • Advise mother how to keep the infant warm on the way to the hospital **Serious bacterial Infection in infants <2 months ^102^:** • Admit to hospital • When possible, do a lumbar puncture and obtain blood cultures before starting antibiotics • For newborns with any signs of serious bacterial infection or sepsis, give ampicillin (or penicillin) and gentamicin as first-line antibiotic treatment* • If at greater risk of staphylococcus infection (extensive skin pustules, abscess or omphalitis in addition to signs of sepsis), give IV cloxacillin and gentamicin • The most serious bacterial infections in newborns should be treated with antibiotics for at least 7–10 days • If an infant is not improving within 2–3 days, change the antibiotic treatment or refer the infant for further management **Meningitis in infants <2 months ^102^:** • The first-line antibiotics are ampicillin and gentamicin* for 3 weeks • Alternatively, give a third-generation of cephalosporin, such as ceftriaxone (50mg/kg every 12 h if <7 days of age and 75mg/kg after 1 week) or cefotaxime (50mg/kg every 12 h if <7 days or every 6–8 h if >7 days of age, and gentamicin for 3 weeks • If there are signs of hypoxaemia, give oxygen • If the infant is drowsy or unconscious, ensure that hypoglycaemia is not present; if it is, give 2ml/kg 10% glucose IV • Treat convulsions (after ensuring they are not due to hypoglycaemia or hypoxaemia) with phenobarbital • Make regular checks for hypoglycaemia *recommended ampicillin dose: 50mg/kg every 12 hours (first week of life) or every 8 hours (weeks 2–4 of life)
Management of children aged 2 months and older
**Septicaemia ^102^** Laboratory: will depend on the presentation but may include • Full blood count • Urinalysis (including urine culture) • Blood culture • Chest X-rays Treatment: • Give IV ampicillin at 50mg/kg every 6 h plus IV gentamicin 7.5mg/kg once a day for 7–10 days; alternatively, give ceftriaxone at 80–100 mg/kg IV once daily over 30–60 min for 7–10 days/ • Give oxygen if the child is in respiratory distress or shock • Treat septic shock with rapid IV infusion of 20ml/kg of normal saline or Ringer’s lactate. Reassess. If the child is still in shock (fluid- refractory shock), start adrenaline or dopamine if available. **Meningitis ^102^** Laboratory: • Confirm the diagnosis with a lumbar puncture and examination of the CSF. If the CSF is cloudy, assume meningitis and start treatment while waiting for laboratory confirmation. • Microscopy should indicate the presence of meningitis in the majority of cases with a white cell (polymorph) count <100/mm ^3^. Confirmation can be obtained from CSF glucose (low: <1.5mmol/litre or a ratio of CSF to serum glucose of <0.4). CSF protein (high>0.4g/litre) and Gram staining and culture of CSF, where possible. • Blood culture if available Treatment: • Ceftriaxone: 50mg/kg per dose IM or IV every 12 hours; or 100mg/kg once daily for 7–10 days administered by deep IM injection or as a slow IV injection over 30–60 min, OR • Cefotaxime: 50mg/kg per dose IM or IV every 6 hours for 7–10 days, OR • When there is no known significant resistance to chloramphenicol and β-lactam antibiotics among bacteria that cause meningitis, follow national guidelines or choose either of the following two regimens: ○ Chloramphenicol: 25mg/kg IM or IV every 6 h plus ampicillin: 50mg/kg IM or IV every 6 h for 10 days, OR ○ Chloramphenicol: 25mg/kg IM or IV every 6 h plus benzylpenicillin: 60mg/kg (100,000 U/kg) every 6 h IM or IV for 10 days.
Prevention of peripartum infections
**Women with group B *Streptococcus* colonization ^100^** • Intrapartum antibiotic administration to women with GBS colonization is recommended for prevention of early neonatal GBS infection (conditional recommendation based on very low-quality evidence) ○ Ampicillin or penicillin G should first be considered for treatment except where there are contraindications (e.g. allergy history) or GBS strain has been microbiologically shown to be penicillin-resistant ○ This recommendation should be implemented within the context of local policy and guidance on screening for GBS colonization. **Women in preterm prelabor rupture of membranes ^100^** • Antibiotic administration is recommended for women with preterm prelabor rupture of membranes (Strong recommendation based on moderate-quality evidence) ○ Erythromycin is recommended as the antibiotic choice for prophylaxis **Women undergoing elective or emergency caesarean section ^100, 151^** • Routine antibiotic prophylaxis is recommended for women undergoing elective or emergency caesarean section. • For antibiotic prophylaxis for caesarean section, a single dose of first-generation cephalosporin or penicillin should be used in preference to other classes of antibiotics.
Managing fever in mothers during pregnancy and labor
**Fever during pregnancy and labor ^116^** Probable diagnosis: cystitis, acute pyelonephritis, septic abortion, amnionitis, pneumonia, uncomplicated malaria, severe/ complicated malaria, typhoid **Septic abortion** • Begin antibiotics* as soon as possible before attempting manual vacuum aspiration **Amnionitis** • Give a combination of antibiotics until delivery - Ampicillin 2g IV every six hours - PLUS gentamicin 5mg/kg IV every 24 hours; - If the woman delivers vaginally, discontinue antibiotics postpartum; - If the woman has a caesarean section, continue antibiotics and give metronidazole 500mg IV every eight hours until the woman is fever-free for 48 hours • If metritis is suspected (fever, foul-smelling vaginal discharge), give antibiotics • If newborn sepsis is suspected, arrange for a blood culture and antibiotics *give ampicillin 2g IV very six hours PLUS gentamicin 5mg/kg IV every 24 hours PLUS metronidazole 500mg IV every eight hours until the woman is fever-free for 48 hours **Chorioamnionitis ^100^** • A simple regimen such as ampicillin and once-daily gentamicin is recommended as first-line antibiotics for the treatment of chorioamnionitis. (Conditional recommendation based on very low-quality evidence)
Managing fever in mothers after delivery
**Fever after childbirth ^116^** Probable diagnosis: metritis, pelvic abscess, peritonitis, breast engorgement, mastitis, breast abscess, wound abscess, wound seroma or wound haematoma, wound cellulitis, cystitis, acute pyelonephritis, deep vein thrombosis, pneumonia, atelectasis, uncomplicated malaria, severe/complicated malaria, typhoid, hepatitis **Metritis** • Transfuse as necessary. Used packed cells, if available • Give a combination of antibiotics until the woman is fever-free for 48 hours: - Ampicillin 2g IV every 6 hours - PLUS gentamicin 5mg/kg IV every 24 hours - PLUS metronidazole 500mg IV every eight hours If fever is still present 72 hours after starting antibiotics, re-evaluate and revise diagnosis **Postpartum endometritis ^100^** • A combination of clindamycin and gentamicin is recommended for the treatment of postpartum endometritis (Conditional recommendation based on very low-quality evidence).

***Pregnant and postpartum women.*** For treatment of chorioamnionitis, the WHO recommends ampicillin and once-daily gentamicin ^100^. A combination of clindamycin and gentamicin is recommended as first-line treatment of postpartum endometritis. Use of intrapartum antibiotic prophylaxis (IAP) to prevent early-onset neonatal disease is described in further detail in section ‘Prevention of perinatal GBS disease through intrapartum antibiotic prophylaxis’.

***Antimicrobial susceptibility.*** Globally, GBS resistance to penicillin G or ampicillin has not been reported. Thus, beta lactams are considered first-line antibiotics for GBS infection or IAP. However, isolates with increased minimum inhibitory concentrations to these antibiotics due to mutations in penicillin binding proteins have been reported primarily from Japan and North America ^137– 141^. Macrolide and/or clindamycin resistant strains have been increasing. There are limited invasive GBS antimicrobial susceptibility data available from LMIC. This largely reflects the relative paucity of invasive neonatal GBS disease surveillance from a majority of LMIC. A systematic review on community-acquired neonatal and infant sepsis in developing countries (Nigeria [4], Iraq [1], Bangladesh [2], India [4], Nepal [1], Tanzania [2], Malawi [1], Uganda [1], Philippines [1], Ethiopia [1], Mozambique [1]) showed that among GBS isolates from neonates, 100% were susceptible to penicillin, 60% (95% CI 25–91%) were susceptible to chloramphenicol, and 65% (95% CI 0–100%) to third-generation cephalosporins ^142^.

## Prevention of perinatal GBS disease through intrapartum antibiotic prophylaxis

### Intrapartum antibiotic prophylaxis

In the 1980s, clinical trials and a large observational study demonstrated that administration of intravenous ampicillin or penicillin during labor to mothers with certain risk factors for GBS transmission was highly effective (efficacy estimates of 80–100%) at preventing invasive early-onset GBS disease ^143– 145^. Effectiveness estimates, although often somewhat lower than estimates from trial settings due to a portion of women receiving less than the optimal prophylaxis duration (at least 4 hours of a beta lactam agent before delivery) or non-beta lactam agents, are consistent with trial findings ^146, 147^. Based on this evidence, penicillin or ampicillin are often the first line agents recommended for prophylaxis, with cefazolin and in narrow instances clindamycin or vancomycin as options for penicillin-allergic women. WHO recommends intrapartum antibiotic administration (first choice penicillin G or ampicillin) to women with GBS colonization based on observed clinical benefits for the neonates (see Table 4); however, the guideline development group acknowledged the challenges in implementing GBS screening and provision of IAP especially in low-resource settings ^100^. WHO recommendations and feasibility in LMIC are further discussed in the two sections below.

### Strategies for targeted intrapartum antibiotic prophylaxis

Because only a portion of women are at elevated risk of transmitting GBS to their infants, universal prophylaxis of all deliveries is not an optimal strategy, particularly since antibiotic exposure is associated with low but non-zero risks. The most immediate risk is maternal anaphylaxis to penicillin which is estimated to occur in four per 10,000 to 4 per 100,000 recipients ^148^. In resource-rich, hospital settings, anaphylaxis-related mortality is exceedingly rare, but in low and middle income countries risks for complications from anaphylaxis, even for hospital births, may be higher. While there is no risk for anaphylaxis in the newborn, due to the very low probability of previous antibiotic exposure and the lack of transfer of maternal IgE antibodies across the placenta, intrapartum antibiotics do impact the microbiome of the maternal birth canal and thus the microbiome acquired by the newborn, particularly for vaginal births. Some studies suggest microbiome alterations, particularly at the time of birth, may result in health impacts well past the newborn period, although these have not yet been substantiated and the risks have not been quantified ^149^.

Two major strategies have been employed to limit the portion of women exposed to intrapartum prophylaxis to those at most risk of transmitting GBS ^9^. The risk-based strategy identifies women for antibiotic prophylaxis based on presence of known risk factors for early-onset disease including maternal fever, prolonged rupture of membranes, preterm delivery, and previous birth to an infant with invasive GBS disease and detection of GBS bacteriuria during the current pregnancy. In different countries employing the risk-based approach, variations may exist in the risk factors screened for, or in the thresholds used to identify risk, based either on local epidemiology or efforts to narrow the portion of women targeted for prophylaxis. Maternal fever is most commonly defined as ≥38°C and prolonged membrane rupture is often ≥18 hours. In contrast, the culture-based screening leads to identification of women with vaginal/rectal GBS colonization late in pregnancy, as a basis for antibiotic prophylaxis indication. Women who present at labor without a culture result are managed according to the risk-based strategy. Variants of the culture-based screening strategy also exist across countries but most recommend screening at 35–37 weeks gestation.

Both strategies have been documented to result in significant declines in invasive early-onset GBS disease, both in single hospitals and population-based analyses, in a range of resource-rich settings ^5^. A population-based comparison of the two strategies in the United States found that the culture-based screening strategy was over 50% more effective than the risk-based strategy, primarily due to the high proportion of GBS positive women who received intrapartum prophylaxis and to the frequency of colonized women without any noted risk factors (18% of the delivering population in the United States) ^150^.

Although current WHO recommendations do not specify a recommended approach for identifying women at risk, antibiotic prophylaxis (erythromycin) to women in preterm pre-labor rupture of membranes is recommended, as part of a strategy to improve the prognosis of infants with preterm birth (strong recommendation based on moderate-quality evidence) ^100^. Antibiotic prophylaxis is not recommended for women in preterm labor with intact amniotic membranes nor for women with pre-labor rupture of membranes at term or near term (36 weeks gestation and above). The latter recommendation is based on the review of evidence from studies in women with duration of ruptured membranes less than 12 hours, and it is acknowledged that there may be a benefit from antibiotic prophylaxis in women with prolonged rupture of membranes (>18 hours) ^100, 151^.

### Feasibility in low- and middle-income countries

Neither of the above strategies were designed in the context of LMIC and both pose implementation challenges, particularly in low-income country settings. In low income countries, safe administration of intravenous antibiotics may not always be affordable or feasible, particularly for settings where births do not occur in hospitals. Even in instances where intrapartum prophylaxis may be feasible, identifying candidates for prophylaxis poses unique barriers. The risk-based strategy has the appeal that the key variables for action can be captured at the time a woman presents for labor. However, even in middle income countries such as South Africa, capture of these variables may prove challenging in a busy labor and delivery setting. For example, in a study of over 8000 deliveries at the main public hospital serving Soweto, South Africa, less than 1% of women were noted as having intrapartum fever suggesting under-ascertainment ^15^. Additionally, gestational age is not always known and clear distinctions between term and preterm deliveries may not always be straightforward. Moreover, because risk factors such as prolonged membrane rupture may evolve over the course of labor, prophylaxis may not always be administered to women who develop risk factors after admission. In resource-rich settings such as the United States, a lower proportion of women with risk factors have been noted to receive prophylaxis compared to GBS-colonized women ^150^; this may prove even more challenging in LMIC where providers care for a higher patient load. Finally, more women in LMIC than in resource-rich settings may present to facilities at a late stage in labor, leaving insufficient time for efficient prophylaxis.

While the risk-based strategy poses challenges, few LMIC are positioned to overcome the implementation and cost challenges associated with late antenatal screening. In particular, LMIC settings rarely have access to a high proportion of women at 35–37 weeks gestation, a strong microbiology laboratory network to process antenatal samples, and systems for effective communication of results to labor and delivery staff.

### Non-vaccine alternatives to intrapartum antibiotic prophylaxis

To date, possible alternative to intrapartum prophylaxis have not proven effective. Chlorhexidine wipes of the birth canal during labor and the newborn at birth were evaluated in a large clinical trial South Africa with no evidence of efficacy against culture-confirmed or clinical neonatal sepsis ^15^. Universal administration of intramuscular penicillin to newborns within 1 hour of birth is implemented at one large center in the United States ^152– 154^; however the lack of a concurrent control makes it difficult to interpret effectiveness or generalizability. This strategy also exposes all newborns to antibiotics. IM penicillin intrapartum does not achieve high enough concentrations rapidly enough, and antenatal use of oral or IM antibiotics have not shown impact ^146, 155– 157^.

## Virulence factors of GBS

GBS disease typically progresses from bacterial colonization, penetration of placental or epithelial barriers, and immune evasion preventing clearance of GBS from the bloodstream. In the case of meningitis, the ability to cross the endothelial blood-brain barrier is also needed ^158, 159^. GBS expresses a number of virulence factors, which play different roles in these steps (summarized in Table 5), but one of the most prominent and best-studied is the capsular polysaccharide (CPS), which protects the bacteria from opsonization and subsequent phagocytosis and intracellular killing ^160, 161^. The type-specific CPS contains a terminal sialic acid, which is identical to a sugar epitope widely displayed on the surface of mammalian cells. Presence of this sialic acid terminal allows GBS to mimic the host cell structure and avoid immune recognition ^111, 162^. To date, 10 different CPS types (Ia and b, and II–IX) have been identified ^161, 163, 164^.

**Table 5.** GBS virulence factors and their role in transition from colonization to invasive disease (adapted from 158).

	Mechanism				
Virulence factor	Colonization	Adhesion	Invasion	Immune evasion	Neurotropism
Fibrinogen binding protein A (FbsA)	+	+			
Fibrinogen binding protein B (FbsB)			+		
Laminin binding protein (Lmb)			+		+
GBS surface adhesion (BsaB)	+	+	(+)		
Alpha C proteins (ACP)	+	+	+	+	
Serine rich repeat proteins (Srr)	+	+	+		
Pili	+	+	+	+	+
Hypervirulent GBS adhesion (HvgA)	+	+	+	(+)	+
β-hemolysin/cytolysin (β-H/C)	+	+	+	+	+
Capsular polysaccharides (CPS)				+	
Streptococcal C5a peptidase of GBS (ScpB)				+	
GBS immunogenic bacteria adhesion (BibA)				+	
Factor H				+	
IgA-binding beta-antigen				+	
D-alanylation				+	
Superoxide dismutase (SodA)				+	

More recently, multilocus sequence typing (MLST) analysis has shown that sequence type (ST) 17 is associated with enhanced invasiveness in neonates independent of capsular serotype although most ST17 isolates are CPS type III ^165^. ST17 displays meningeal tropism, and has been referred to as the hypervirulent clone ^166^.

It has been hypothesized that the GBS isolates causing invasive GBS disease in neonates worldwide emerged from a few successful clonal lineages, and virulence factor identification to date has focused on elements common across these clones ^161^. Factors under investigation to date include the C5a peptidase, the AlphaC-like surface protein family, the Sip-protein, and pilus islands, all of which have different roles in the infection process ^161^, and have been investigated as vaccine targets. Pili mediate GBS resistance to cationic antimicrobial peptides (AMPs), which are components of the host innate immune system that play a critical role in combating bacterial infections ^167^, and also facilitate adherence and attachment of the pathogen to host mucosal cells. More recently, a surface-anchored adhesion protein called hypervirulent GBS adhesion (HvgA) was identified from comparative expression analysis between clones of different virulence. HvgA is considered to be a specific virulence factor of hypervirulent ST17 ^168^. In a manner similar to that of pili, HvgA mediates both colonization and invasion in the intestine, which appears to be a prerequisite for meningitis in the neonatal mouse model ^161^.

## GBS vaccine development

### Biological rationale for a vaccine

Prevention of neonatal GBS disease has been the primary focus for GBS vaccine development. Most cases of early-onset neonatal and young infant disease occur within the first 24 hours. Therefore, maternal immunization rather than direct vaccination of newborns is required to prevent neonatal and young infant disease. In animal models, passive immunization ( *e.g.*, transferring sera of animals exposed to GBS disease) and active immunization ( *e.g*., mouse maternal vaccination-neonatal pup challenge model) have been shown to be protective against development of GBS disease (see below for details on animal models). In humans, transplacental transfer of protective maternal antibodies against GBS was first reported by Baker and colleagues ^169^. Their study showed that mothers whose infants developed invasive GBS disease from serotype III had significantly lower levels of serum IgG levels to CPS III compared to mothers whose infants were exposed to type III but did not develop disease. Subsequent studies reported similar findings with other GBS serotypes ^170, 171^ and the association of low maternal GBS CPS specific IgG levels and the risk of GBS disease in their infants was further described ^19^. Attempts have been made to identify a threshold that would confer protection against GBS disease for vaccine development.

Maternal IgG is transmitted transplacentally to the fetus, mediated by the neonatal Fc receptor (FcRn): maternal IgG is endocytosed within placental syncytiotrophoblast cells and bound to FcRn in the acidic environment of the endosome, then released to the fetal side of the syncytiotrophoblast when exposed to physiological pH ^172, 173^. Results from phase I/II trials have shown that maternal immunization with glycoconjugate vaccines results in increased CPS-specific antibody levels in the infants which persists for about 2 to 3 months ^174, 175^. Transfer of maternal IgG initiates at around 17 weeks gestation, although most of the transfer occurs after 33 weeks gestation ^176^. Therefore, infants born before 34 weeks gestation are likely to have decreased amounts of IgG ^177^.

A study by Baker *et al*. using data from the United States estimated that third trimester maternal immunization could prevent approximately 46% of all late-onset GBS infections, given that 63.3% of infants who developed late-onset disease were born ≥35 weeks gestation, and of those born ≥35 weeks gestation, approximately 72% of infections occurred during the first 6 weeks of life ^176^. This is based on the assumption that infants born ≥35 weeks gestation would have acquired sufficient concentrations of maternal antibodies, which would protect the infant from GBS disease for the first 6 weeks of their life (translating to two half-lives of antibody decay). The optimal timing of maternal immunization that would maximize protection against young infants requires further investigation.

Results from a phase II randomized controlled trial have shown that the III-TT vaccine delayed the acquisition of vaginal and rectal GBS III (NCT00128219) ^178^. Another study reported an association between increased serum CPS IgG levels and reduced homotypic GBS rectovaginal acquisition ^179^. If the vaccine reduces maternal colonization, maternal vaccination could further reduce the risk of neonatal disease by reducing exposure to GBS in the first months of life.

### Newborn and young infant response to natural GBS infection

Opsonization, followed by phagocytosis (ingestion of invading microorganism) and intracellular killing are the main mechanisms of host defense against GBS infection ^180^. Opsonization requires the deposition of specific antibody and complement on the bacterial surface, and antibody and complement do not kill GBS in the absence of phagocytes ^181^. Type III GBS-CPS was shown to prevent activation of the alternative complement pathway but this effect can be overcome by the presence of a sufficient amount of CPS antibody ^182, 183^.

Immaturity of the immune system makes neonates more susceptible to infections: neutrophils have a small storage pool at birth, and are less responsive to chemoattractants than later in life. Neonatal monocytes, which mature into macrophages, are impaired in their capacity for killing intracellular GBS ^184^. Newborns have an impaired ability to form antibodies in general, and are particularly deficient in their ability to mount antibody responses against polysaccharide antigens ^185^. Altogether, their capacity for GBS CPS antigen-specific protection is determined largely by the placental transfer of maternal IgG antibodies ^186^. Therefore, the goal of maternal immunization is to induce GBS-specific antibody levels in the mother to achieve antibody levels in the child that would confer protection during the first 3 months of life.

### Animal models

***GBS disease models.*** Pre-clinical studies using animal models are important to obtain sufficient data on safety, immunogenicity and potential efficacy of candidate vaccines before proceeding to clinical trials. A wide range of animal models has been used to study GBS-host interactions and to provide means to test potential therapies and vaccine approaches. A sampling, rather than a comprehensive review, is provided below.

Mice have been commonly used to model GBS infections. The earliest animal models studies of GBS infections date to the 1930s ^122^. In later studies, intraperitoneal or intravenous models of GBS infection in adult or neonatal mice were developed to simulate human infections ^187, 188^. In some cases, oral inoculation has been used as a means of inducing systemic infection in mice ^189^. Notably, in both mice and rats, there appears to be an age-related decrease in susceptibility to invasive GBS infection ^190, 191^.

A large number of other animal model systems have been explored, including chicken embryo ^192^, rabbits ^193^, sheep ^194– 196^, piglet ^197^, and non-human primates ^198, 199^. Some of these models (especially the large animal models) have been used to provide insights that are difficult or impossible to study in mice. The sheep ^194– 196^ and piglet models ^197^ are of particular relevance for the study of hemodynamic changes in host animals during GBS sepsis. Non-human primate models of GBS infection have been used sparingly, but they are of particular utility in modeling newborn infections and host responses *in vivo* ^198, 199^.

***Animal models of GBS colonization.*** Fewer studies have used animals to model asymptomatic GBS carriage, despite the importance of the carrier state for maintenance of GBS in the population and the role of maternal colonization as the major risk factor for neonatal disease. Most recent work in this area has used murine models of vaginal or gastrointestinal colonization.

Vaginal colonization models have allowed determination of specific bacterial or host factors involved in carriage in the absence of invasive disease ^200– 208^. Gastrointestinal GBS colonization has been modeled in gnotobiotic mice and used as a means to understand the role of surface proteins in GBS carriage ^209^. Neonatal mice have also been used as a model for gastrointestinal carriage, especially as a prelude to invasive disease ^168^. Oral colonization of infant rats has been used to examine the utility of antibiotics to decrease mucosal bacterial load ^210^.

***Animal models of ascending infection and/or perinatal GBS transmission.*** To examine the role of ascending infection in adverse pregnancy outcomes ( *e.g*., preterm delivery, stillbirths), animal models simulating human infections have been explored. Examples include intracervical GBS inoculation of rabbits ^211– 213^, murine intravaginal/intrauterine/intraperitoneal inoculation ^214– 216^; catheterization and intraamniotic instillation of GBS has been used to model chorioamnionitis in non-human primates and to study its effects on fetal lung tissue ^217– 220^. However, initial attempts to create an ascending infection animal model secondary to chronic vaginal colonization, which is a better simulation of human infection, were unsuccessful ^213^. Recently, Randis and colleagues have developed a model of GBS ascending infection during pregnancy secondary to vaginal colonization using pregnant mice. This model may shed light on the role of bacterial virulence factors such as beta-hemolysin/cytolysin in causing adverse pregnancy outcomes associated with maternal GBS colonization ^203^.

***Preclinical studies of GBS vaccines in animal models***

**a. Passive immunization** Animal models have been used to examine the effect of antibody delivery (passive immunization) on invasive GBS disease *in vivo*. The first studies used generation of antibodies in rabbits followed by passive protection of mice exposed to systemic GBS infection ^122, 221^. Subsequent studies used hyperimmune serum or purified antibody preparations to provide protection to neonatal experimental animals ^199^.

**b. Active immunization** Active immunization formulations used in experimental animals have included whole killed GBS, purified bacterial components (generally capsular polysaccharide or purified proteins), or conjugate vaccine preparations. Most studies have used systemic vaccination (intramuscular, subcutaneous, or intraperitoneal), though some investigations of mucosal vaccine delivery (intranasal, oral, rectal, or intravaginal) have been reported ^222– 227^.

Most vaccine studies have used protection from invasive infection as their outcome, but reports of potential effects of vaccination on GBS colonization have also been presented ^228^. The most frequently used model to evaluate the efficacy of GBS vaccines is the mouse maternal vaccination-neonatal pup challenge model. In this model, female mice are actively vaccinated, and their offspring are challenged with GBS ^229, 230^. Maternal IgG is transferred to the pups similar to maternal antibody transfer in humans, and most pups of mothers vaccinated with a range of conjugate formulations have survived challenge ^231, 232^. This model continues to be used to test new vaccine targets ^233– 235^. However, GBS strains isolated from human infections may be highly adapted to their human host, and results obtained from mouse models must be interpreted with caution ^236^. For example, human GBS isolates may express surface proteins that specifically interact with the human hosts but not with other animals ^236– 238^. In addition, the shorter gestational period of mice (19–22 days) should be taken into account to measure the timing of vaccination and passive protection in neonates ^239^.

The structure and function of antibodies induced by vaccination and the kinetics of maternal antibody transfer to the fetus are most similar between human and non-human primates. Baboon models have been used in preclinical GBS vaccine studies ^229, 230, 240^. As in mouse models, these studies showed that GBS conjugate vaccine induced CPS-specific antibodies ^230, 240^, and there was a correlation between maternal and infant baboon serum antibody levels ^230^. Differences have been noted in the kinetics of antibody responses and waning between humans and baboons ^241^.

### History of GBS vaccine development

***Polysaccharide vaccines.*** GBS capsular polysaccharide (CPS) has been the primary target for vaccine development. In the 1930s studies demonstrated that CPS-specific rabbit sera could be used to protect mice against lethal challenge with GBS ^242^. The first purified type III CPS vaccine underwent phase I testing in healthy adults in 1978 ^243^, and subsequently type Ia and II CPS vaccines were tested. Type II CPS was found to be the most immunogenic, while type Ia and III showed an immune response in about half of the recipients ^244^. Most adults (nearly 90%) had very low serum concentrations of CPS specific antibodies before immunization, which was considered to indicate immunologic naivety to GBS polysaccharides, and was a partial predictor for a poor immune response ^244, 245^. Favorable safety of CPS vaccines was shown on a small scale in non-pregnant adults and among pregnant women ^245, 246^, and infant antibody levels in cord serum correlated with maternal antibody levels at delivery ^246^.

***Glycoconjugate vaccines.*** Immunogenicity of polysaccharides is enhanced by covalent conjugation with a carrier protein. Glycoconjugate vaccines have been developed for *Haemophilus influenzae* type b (Hib), *Neisseria meningitidis* and *Streptococcus pneumoniae*. Unlike T-cell-independent B-cell activation by non-conjugated polysaccharide antigens, glycoconjugate vaccines have the potential to induce both B- and T-cell memory and produce a stronger and highly functional IgG response through antibody class switching ^160^.

The first GBS glycoconjugate vaccine trial conducted in humans involved a GBS III CPS-tetanus toxoid (III-TT) glycoconjugate ^160, 247^. Healthy non-pregnant women were recruited and randomized to receive III-TT, type III CPS vaccine, or placebo ^247^. Results showed that the highest dose of III-TT produced higher levels of type III CPS-specific antibody measured two weeks after vaccination, and that the proportion of recipients achieving a ≥4-fold rise in antibody concentration was higher among those who received III-TT compared to those who received unconjugated type III CPS vaccine ^247^, suggesting that the glycoconjugated vaccines are able to induce a more robust immune response compared to polysaccharide-only vaccines. Following this first trial, phase I trials of monovalent Ia, Ib, II and V-TT conjugates showed immunogenicity of a single dose suggesting no need for addition of an adjuvant ^241, 248, 249^. In another randomized controlled study in healthy non-pregnant women, receipt of GBS III-TT was associated with protection against future acquisition of type-specific GBS colonization, with 36% vaccine efficacy for vaginal acquisition and 43% efficacy for rectal acquisition compared to controls who received tetanus and diphtheria toxoids (clinicaltrials.gov NCT00128219) ^178^.

To achieve broader coverage against the GBS serotypes causing disease in humans, several multivalent vaccines have been developed and tested in humans. The immune response in subjects who received a bivalent vaccine containing II-TT and III-TT glycoconjugates did not differ statistically from the antibody responses to monovalent vaccines ^250, 251^. Novartis (now GSK) has developed a trivalent (serotypes Ia, Ib, III) glycoconjugate vaccine conjugated to a CRM _197_ carrier and conducted several phase I and II clinical trials in healthy non-pregnant and pregnant women (clinicaltrials.gov NCT01150123, NCT01193920, NCT01446289).

Initially, GBS vaccine studies have used tetanus toxoid (TT) as the carrier protein, but there have been concerns about possible immune interference and adverse events upon subsequent vaccination with TT-conjugated vaccines ^229, 250^. CRM _197_, a nontoxic mutant of diphtheria toxin (DT), is another carrier protein, and has been used in the investigational trivalent GBS vaccine that went through phase II studies (clinicaltrials.gov NCT01412801, NCT01446289) ^174, 252^. A study showed equivalent immunogenicity of CPS V-TT and CPS V- CRM _197_ against the target GBS antigen ^249^.

While vaccines conjugated to TT (used in Menitorix ^®^[MenC-TT/PRP-TT, GSK]) and DT (used in Menactra ^®^[Men ACWY-DT, GSK] and in Synflorix ^®^[PCV10, GSK]) have shown to induce immunity against TT or DT, vaccines using CRM _197_ as the carrier protein (used in Prevnar13 ^®^[PCV13, Pfizer], Menveo ^®^[MenACWY-CRM, GSK], investigational trivalent GBS vaccine [GSK]) have not shown to induce immunity against DT ^174, 253^. The interest of inducing protection against tetanus through a TT-conjugated GBS vaccine could be considered where maternal and neonatal tetanus remain a concern.

Lastly, there have been concerns that use of CRM _197_ may interfere with responses to routine infant vaccines that use CRM _197_ as the carrier protein, such as PCV13, Hib, and MenACWY ^254– 256^. In addition, use of CRM _197_ may interfere with responses to routine diphtheria vaccination in infants, but results from a phase II study did not show any evidence of interference ^174^.

***Protein-based vaccines.*** Polysaccharide-based vaccines typically only provide protection against CPS types included in the vaccine or closely related serotypes, and may be vulnerable to serotype replacement/switching. Therefore, efforts have been made to identify proteins common to all GBS as the basis of a vaccine that would confer broad protection against GBS ^250^.

Until whole genome sequences of two GBS strains became available in 2002, only a limited number of proteins involved in GBS pathogenesis were identified as potential vaccine candidates ^250^. Rib and alpha are among the GBS surface proteins that have been studied extensively as possible vaccine targets ^257, 258^. Recently, MinervaX, a privately held Danish biotech company, has initiated phase I clinical trials with a protein vaccine based on a fusion of the N-terminal portion of two surface proteins, AlphaC and Rib (GBS-NN) (NCT02459262) ^259^. MinervaX expects that GBS-NN will protect against up to 95% of GBS isolates, given the broad expression of AlphaC and Rib as well as cross-reactive proteins ^259^.

During the past decades, the application of recombinant DNA techniques and the availability of complete bacterial genomes have allowed use of genome-based vaccinology to identify new protein vaccine candidates ^250^. Investigators from GSK used reverse vaccinology to identify a conserved sequence encoding components of pili proteins on the bacterial surface. A vaccine based on a combination of these proteins conferred protection against different GBS strains in a mouse model ^260^. However, coverage against all GBS strains was not possible due to antigenic variation associated with the pilin subunits ^250, 260^. Structural vaccinology was successfully applied to design an optimized BP-2a protein, a subunit of the backbone protein of the GBS pili known to have high gene variability ^250^. The protective capacity of a BP-2a variant is restricted to a small region (D3), and each variant fused into a single recombinant chimeric construct expressed in *Escherichia coli* which conferred strong protection against all six strains expressing a BP-2a variant in challenged mice ^235^.

### Evidence for immune correlates of protection

***GBS-specific antibody concentration and correlates of protection.*** Sero-epidemiological studies showed some evidence in favor of an association between low maternal GBS CPS specific IgG levels and the risk of GBS disease in offspring. Associations between maternal GBS surface-protein antibody concentrations and invasive disease in their infants have not been as clearly established: among the surface proteins studied so far (surface immunogenic protein [Sip], resistance to proteases immunity group B [Rib], AlphaC protein, BetaC protein, fibrinogen-binding protein A, GBS-immunogenic bacterial adhesion, and pilus-island surface protein antibodies), limited data suggest that antibodies against alphaC and Rib proteins may provide protection against invasive neonatal GBS disease ^258, 261– 265^.

***Evidence from sero-epidemiological studies.*** Most of the earlier studies comparing capsular antibody concentrations between cases and controls were done using a small sample size ( *e.g*., ≈10–50 cases total per capsular serotype). More recent studies with larger sample sizes ( *e.g*., >50–300 cases total per capsular serotype) have attempted to identify a serotype-specific IgG level in mothers that would confer protection against infant disease due to the same serotype ^266– 269^. A summary of studies published after 2000 is shown in Table 6. Both studies by Lin and colleagues were case-control studies using data collected from multiple study sites in the United States ^266, 269^. Maternal and cord serum samples were collected from enrolled participants after delivery and antibody levels were compared between cases (neonates who developed early-onset disease and their mothers) and controls (neonates who remained healthy despite being colonized with the same serotype and their mothers). The case-control study by Baker and colleagues was also a multi-center study in the United States and compared maternal serum samples from cases (those whose infants developed early-onset disease due to specific serotypes) matched by age and ethnicity with those from controls (those who were colonized with the sample capsular serotypes but whose infants did not develop disease) ^267^. The study by Matsubara and colleagues was conducted at a single institution in Japan and compared serum antibody levels of pregnant women with serotype VIII colonization with stored serum samples from four mother-and-neonate pairs with early-onset serotype VIII infection ^270^. Dangor and colleagues conducted a matched case-control study in South Africa; cases were infants with laboratory-confirmed invasive GBS disease within <90 days of age, and controls were age-matched healthy infants, whose mothers were colonized with the same GBS CPS serotypes as cases. Maternal and infant serum from cases were compared with those of controls (or cord serum in case of controls of early-onset disease ^268^.

**Table 6.** Studies published since year 2000 describing capsular antibody concentrations in mothers of infants with and without invasive group B *Streptococcus* disease (adapted from Dangor *et al.* ^272^).

Study Country Study design	Case ^1^ serotype (n)	EOD	LOD	Control ^2^ serotype (n)	Assay (GBS antibodies measured)	Antibody levels (μg/ml) in cases with invasive GBS disease		Antibody levels (μg/ml) in healthy infant controls		Suggested threshold	GBS risk reduction at suggested threshold	
						Mother (n)	Newborn/ infant (n)	Mother (n)	Newborn/ infant (n)		Measurement	Value
Lin *et al.* (2001) USA CC	Ia (53)	53	0	Ia (336)	ELISA Ia (IgG)	0.32 ^3^ (49)	0.22 ^3^ (49)	0.65 ^3^ (326)	0.52 ^3^ (323)	≥5μg/ml	OR (95% CI)	0.12 (0.02–0.93) ^4^
Lin *et al.* (2004) USA CC	III (29)	29	0	III (330)	ELISA III (IgG)	2.73 ^3^ (28)	2.03 ^3^ (27)	4.27 ^3^ (306)	3.29 ^3^ (312)	≥10μg/ml	OR (95% CI)	0.09 (0.01–0.78) ^5^
Matsubara *et al.* (2002) Japan Cohort	VIII (4)	4	0	VIII (13)	ELISA VIII (IgG)	0.41 ^3^ (4)	0.49 ^3^ (4)	5.53 ^3^ (13)	NR	>1μg/ml	OR (95% CI)	<0.001 ^6^
Baker *et al.* (2014) USA CC	Ia (17) III (9) V (7)	33	0	Ia (51) III (27) V (21)	ELISA Ia, III, V (IgG)	0.20 (0.06– 1.68) ^3^ (17) 0.06 (0.02– 0.12) ^7^ (9) 0.09 (0.04– 0.80) ^7^(7)	NR	1.83(0.20 –5.54) ^3^(51) 1.64(0.14– 5.51) ^7^ (27) 0.53(0.07– 1.0) ^7^(21)	NR	>1μg/ml ^8^ (for all serotypes)	OR (95% CI) ^8^	0.11 (0.01–0.74) ^8^ 0.09 (0.00–0.72) ^8^ 0.29 (0.01–3.10) ^8^
Dangor *et al.* (2015) South Africa CC	Ia (27) III (29)	22	34	Ia (43) III (31)	Luminex Ia, III (IgG)	0.05(0.02– 0.24) ^7^ (27) 0.14 (0.08– 0.33) (34)	0.01(0.01– 0.07) ^7^ (27) 0.04(0.02– 0.08) ^7^ (34)	0.29 (0.06– 1.60) ^7^ (29) 0.29 (0.13– 0.58) ^7^ (34)	0.19 (0.05 –1.54) ^7^ (29) 0.15 (0.06 –0.44) ^7^ (34)	≥6μg/ml ≥3μg/ml	Risk of GBS disease at threshold (Bayesian model)	6.5% (credible interval: 1.5–21.9) 1.3% (credible interval: 0.1–9.9)

This table was adapted with permission from Taylor & Francis ^272^. CC: case-control; EOD: early-onset disease; LOD: late-onset disease; NR: not reported; OR: odds-ratio ^1^ Case, number of infants with invasive disease stratified by disease serotype ^2^ Control, number of GBS rectal/vaginal/cervical colonized mothers of healthy mothers of healthy infant stratified by colonizing serotype ^3^ Geometric mean concentration ^4^ Adjusted for maternal age <20 years old, primigravida, diabetes during pregnancy, insulin required during pregnancy, membranes ruptured ≥12 h before delivery, and delivery by Caesarean section, in the multivariable regression models ^5^Adjusted for female sex of neonate, performance of invasive procedure(s) during labor, intrapartum antibiotics, maternal fever, and delivery by Caesarean section ^6^None of the cases had an antibody level above the threshold ^7^Median (interquartile range) ^8^The suggested threshold was >1 μg/ml but OR of early onset GBS disease in neonates was calculated using maternal serum capsular polysaccharide-specific IgG concentrations at delivery of ≥0.5μg/ml compared to those with <0.1 μg/ml in a logistic regression model

The results showed that in general, there was an inverse relationship between maternal serotype-specific IgG levels and the risk of their infants developing GBS disease ( Table 6. Except for the studies by Baker and Dangor ^267, 268^, all studies used relative statistical measures to estimate thresholds, whereas the study by Baker and Dangor used Bayesian modeling to determine the threshold. This method is considered to be robust using small sample sizes and does not depend on preselected reference values ^271^. Only the study by Dangor *et al*. included late-onset disease and due to the small sample size, they were not able to assess correlates of protection separately for early-onset disease and late-onset disease ^268^.

***Functional antibody concentrations and other potential endpoints of relevance.*** While the above studies showed evidence of an association between antibodies and risk of invasive infection, some infants developed disease despite having high antibody levels. Measurement of functional antibodies rather than overall antibody concentrations may be important to shed further light on immune correlates of protection, as total antibody levels might include inactive antibodies ^272– 274^. An example of this is the opsonophagocytosis killing assay (OPkA) ^182, 275^, which mimics the *in vivo* process of the killing of the bacterium by host effector cells following opsonization by specific antibodies. Antibody-mediated bacterial killing has also been shown to protect infants from GBS disease and may be a more useful marker than purely measuring antibody quantity via an enzyme-linked immunosorbent assay (ELISA)-type assay ^267^. Functional antibody assessed by OPkA appears to correlate more closely with protection from GBS colonization, a precursor to disease in infants, than CPS-specific antibody concentration ^276^. However, OPkA assays are laborious to perform and require large volumes of test sera. This is a critical issue in studies where sample volume is at a premium, such as in neonatal studies. Other assays, including an antibody-mediated complement C3b/iC3b deposition assay ^28, 277^ have been developed that are less labor intensive and less variable as they do not rely on human phagocytes and require small serum volumes. Avidity assays have also been explored but results indicate no significant difference in median avidity between antibodies induced by unconjugated or conjugated vaccines with a large range of values obtained for both vaccines ^278^.

### Status of assay and reagent standardization efforts

Different assay methods, antigen constructs and standard quantitation for serotype-specific antibody levels ^267, 269^ have made comparison across studies challenging ^272^. Different specific antibody concentrations that could be associated with protection from disease have been defined. However, these vary across studies and by GBS serotypes ( Table 6), and there has been significant controversy regarding appropriate laboratory methods to derive such thresholds reliably ^279^.

Historically, the radioantigen binding assay (RABA) has been seen as the gold standard for the quantification of anti-GBS antibody as it measures antibody in its native state ^169^. However, the RABA has low sensitivity towards the lower limit of quantification and is unable to identify immunoglobulin of different isotypes and subclasses as so offers an incomplete picture of immunoglobulin concentration. Several more sensitive isotype-specific ELISA have subsequently been developed and have been used in the majority of vaccine studies to date; however, the estimated antibody concentration required to reduce the risk of GBS disease varied ^266, 267, 269, 270, 280– 284^. These assay methods vary, resulting in difficulties in extrapolating data between studies. More recently, studies have used Luminex or Bioplex platforms in order to improve the sensitivity and throughput of these assays and allow multiplexing. However, none of these ELISA or Luminex assays provide information on the ability of the antibodies to neutralize GBS. Therefore, an ELISA alone may not be sufficient in predicting protective immunity from GBS infection ^272^. A possible solution to this may be the development of an effective functional antibody assay that could be used as an *in vitro* correlate of protection, such as OPkA.

However, to achieve this goal for GBS, assay standardization is required for each GBS antigen of relevance and for each serotype ( Table 6. It is also possible that proposed thresholds might vary depending on study population differences ( *e.g*., higher prevalence of HIV positive patients in the study ^268^). Efforts to standardize quantitative and functional immunoassays are needed for phase II and phase III GBS vaccine studies using immunogenicity endpoints.

### Vaccine development pathway

The development of a GBS vaccine as considered here is unique in that the primary target population is pregnant women, as opposed to vaccines that WHO currently recommends in pregnant women ( *e.g*., tetanus toxoid, inactivated trivalent influenza vaccine, acellular pertussis vaccine) which were not developed nor licensed to target pregnant women ^285– 287^. The anticipated vaccine development pathway will likely begin with preclinical studies relying on animal models to assess the immunogenicity and safety of the product. Potential adverse outcomes in both mothers and their offspring are evaluated, including reproductive and developmental toxicity associated with the product ^288^. Upon favorable pre-clinical evaluation, first time in human studies are conducted in healthy adults. Phase I testing could start in non-pregnant women of childbearing age, in a limited number of participants ( *e.g*., <100) ^288^. Phase II studies of up to several hundred subjects per trial typically provide more information on common local and systemic reactions and immunogenicity evaluations of dose range and dose schedule ^288^. Evaluation in pregnant women would typically only start upon favorable evaluation in non-pregnant women. In addition to adding to information on adverse events among mothers, phase II trials in pregnant women can provide initial information about safety effects in newborns, as well as information about IgG antibody transfer ratios to the newborn and duration/decay of these antibodies over time (see following section on endpoints of relevance in immunogenicity studies). Phase III trials would typically have a large enough sample size to provide data supportive of licensure ^288^. Phase III pivotal licensure studies most classically include a well-defined primary clinical endpoint, but alternative pathways to licensure are being discussed in the case of GBS vaccines, considering the possibility of establishing a regulatory acceptable immune correlate of protection. Post-licensure evaluations may play a critical role in characterizing rarer safety events and effectiveness under real-world conditions, as well as in special populations of interest.

### Safety of vaccination during pregnancy

Vaccines targeting maternal immunization during pregnancy must demonstrate favorable safety for the mother, the developing fetus and the newborn. Upon request by the WHO Strategic Advisory Group of Experts (the senior WHO vaccine governance board), the WHO Global Advisory Committee on Vaccine Safety (GACVS) recently reviewed safety data on existing vaccines for maternal immunization in pregnancy ^289, 290^. The GACVS concluded that there is no evidence of adverse pregnancy outcomes from the vaccination of pregnant women with currently licensed inactivated virus, bacterial, or toxoid vaccines. They concluded that pregnancy should not preclude women from immunization with these vaccines if medically indicated. As described in the previous paragraph, WHO currently recommends administration of tetanus toxoid, inactivated trivalent influenza vaccine, and acellular pertussis vaccine to pregnant women, although none of these vaccines were licensed for use in pregnant women ^285– 287^. Conjugate vaccines (either licensed or investigational), when conjugated with different carrier proteins ( *e.g*., TT, DT, CRM _197_), as well as vaccine formulations including alum and oil-in-water emulsions as adjuvants have been used in pregnant women, and favorable safety has been documented ^174, 252, 291, 292^. Further considerations on safety evaluation of GBS vaccine candidates are presented in following sections.

## Current GBS vaccine candidates in development

### Review of existing candidates

CPS-based vaccines have been the most extensively studied among vaccine candidates, and trivalent glycoconjugate vaccine candidates have gone through phase I and II trials. Currently, there are no plans for these trivalent vaccine candidates to move on to phase III studies.

GBS protein vaccines using other target antigens ^293^ and polysaccharide vaccines conjugated with different carriers ( *e.g*., GBS80 pilus protein, peptide) ^233, 294^ have been tested in animal models. GBS-NN is undergoing phase I evaluation (NCT02459262). A summary of candidate vaccines is shown in Table 7.

**Table 7.** Development status of current vaccine candidates (adapted from 329).

Developer	Candidate name/identifier	Preclinical	Phase I	Phase II	POC	Phase III
NIH	Tetanus toxoid-CPS conjugates: monovalent (multiple studies), bivalent (one study); CRM _197_-CPS conjugate: monovalent (one study)	X	X	X	X (trial in pregnant women)	
Novartis/GSK	CRM _197_-CPS conjugate: monovalent (multiple), trivalent (several)	X	X	X	X (trial in pregnant women)	
Minervax	N-terminal domains of the Rib ad AlphaC surface proteins	X	X			
Novartis/GSK	Pilus proteins	X				
Various academic groups	Other protein(s) and/or protein-CPS conjugates	X				

CPS: capsular polysaccharide, GSK: GlaxoSmithKline, NIH: National Institutes of Health, POC: proof of concept

### Safety data from phase I and II studies

***Non-pregnant women.*** Multiple polysaccharide and protein conjugate GBS vaccines have been tested in healthy non-pregnant women, although the number of volunteers included was usually small ( *e.g*., ≤30 in each vaccine group). Earlier studies testing vaccine dose-response have shown local pain or mild redness which seemed to be more frequent upon immunization with higher doses ^241, 247, 248^. More recently, a phase Ib randomized, observer-blind and placebo-controlled trial of a trivalent (serotypes Ia, Ib, III) GBS CPS-CRM _197_ conjugate vaccine was conducted among healthy non-pregnant women (NCT01150123) ^295^. In this study, approximately 40 women were enrolled in each vaccine group, which consisted of different dosing schedule ( *e.g.*, one dose vs. two doses) and different use of adjuvants (no adjuvant, use of Al(OH) _3_, or MF59 [either half dose or full dose]). Results showed that local reactogenicity was increased in those who received vaccines with adjuvants (range: 40–42% in placebo group, 75–88% in vaccine group without adjuvants, 93–100% in those with Al(OH) _3_, 83–100% in those with half dose MF59, and 93–100% in those with full dose MF59); the proportion of solicited systematic reactions was less frequent (58–65% in the placebo groups, 50–85% across vaccine groups). Serious adverse reactions were similar among the vaccine and the placebo groups (5–11% in placebo group, 0–5% in vaccine group without adjuvants, 0–15% in those with Al(OH) _3_, 0–8% in those with half dose MF59, and 5–15% in those with full dose MF59), but none of them were considered related to vaccination, and there were no deaths or premature withdrawals due to adverse events (NCT01150123) ^295^.

***Pregnant women and newborns.*** The first phase I trial that used a glycoconjugate vaccine among pregnant women was conducted with III-TT vaccine with a saline placebo control group ^175^. A total of 30 participants were enrolled, and no vaccine-associated serious adverse events were observed. Mild to moderate pain at the injection site occurred in 70% of the vaccine recipients compared to 40% in placebo recipients; 10% had redness at the injection site in the vaccine group compared to 0 in the placebo group. Obstetrical complications, mostly related to need for cesarean section or postpartum fever, occurred in 35% of vaccine and 70% of placebo recipients. All neonates had an uncomplicated hospital course in both groups. Results from a phase II randomized, observer-blind, multicenter study using trivalent (Ia, Ib, III) GBS polysaccharide-CRM conjugate vaccine among pregnant women has been published recently (NCT01446289) ^174^. A total of 86 women at 24–35 weeks gestation were enrolled, of whom 51 were assigned to the vaccine group. Reports of solicited adverse reactions were similar between the groups, with 54% of the vaccine group vs. 53% in the placebo group reporting at least one solicited reaction. Reported rates of systematic reactions were similar, although more participants in the vaccine group reported local adverse reactions (40% in the vaccine group vs. 24% in the placebo group). All women gave birth to single, live born neonates, and obstetric outcomes were similar between the two groups. No infant deaths occurred during the study period, and serious adverse events were reported in 24% of the vaccine and 31% of the placebo group infants.

### Immunogenicity

***Endpoints of relevance.*** The phase I/II trials using investigational trivalent GBS conjugate vaccines quantified GBS serotype-specific antibody levels using ELISA and reported as geometric mean concentrations (GMC) (NCT 01446289, NCT01150123, NCT01412801). None of these studies evaluated antibody functionality, but earlier GBS conjugate vaccine studies reported Opsonophagocytic assay (OPA) evaluation ^175, 241, 247, 248^. An ongoing phase I trial of GBS-NN is using both ELISA and OPA to measure immunogenicity.

***Evidence from Phase I and II trials***

**a. Non-pregnant women (NCT01193920, NCT01150123)** A phase Ib/II trial in which 40 non-pregnant women received two doses of trivalent GBS vaccine (Ia, Ib, III, 20/20/20μg) showed that compared to the placebo group, the geometric mean concentration (GMC) of antibody measured by ELISA a month after the second vaccination was significantly higher for all measured serotypes (serotype Ia 40 μg/mL in vaccine group vs. 0.88 in placebo group; serotype Ib 5.3 vs. 0.25; serotype III 11 vs. 0.61), and remained higher a year after the first dose (serotype Ia 15 μg/mL in vaccine group vs. 0.86 in placebo group; serotype Ib 5.28 vs. 0.4; serotype III 7.03 vs. 0.3) (clinicaltrials.gov: NCT01193920) ^296^. In a study by Leroux-Roels and colleagues comparing vaccine groups with different antigen concentration, adjuvants, and dosing schedule (NCT01150123) ^295^, results showed that all vaccine groups had a higher GMC compared to placebo groups at both 61 days and 361 days after vaccination; a higher dose level, the presence of aluminum hydroxide adjuvant or a second dose did not significantly increase antibody concentration. The exception was a higher GMC against serotype III one year vaccination in the group having received a second dose. When stratified by antibody concentrations at baseline, women who had undetectable antibody concentrations had lower antibody responses than those with detectable antibodies at baseline.

**b. Pregnant women and newborn (NCT01446289)** The aforementioned phase I trial using III-TT vaccine in pregnant women reported that 19 of 20 recipients had 4-fold increases in III CPS-specific IgG after vaccination relative to pre-vaccination levels, infant cord levels were approximately 70% of maternal values at delivery, and opsonophagocytic killing measured in sera of infants born to vaccine- but not placebo-recipients persisted until 2 months of age, suggesting the potential to protect against both early- and late-onset GBS infant disease ^175^. A phase II placebo-controlled trial using a single dose of trivalent (Ia, Ib, III, 5/5/5μg) GBS polysaccharide-CRM _197_ conjugate vaccine administered to pregnant women at 24–35 weeks gestation was conducted in Belgium and in Canada (NCT01446289) ^174^. Levels of antibodies against serotypes Ia, Ib, and III at delivery were respectively 16-, 23- and 20-fold higher than pre-vaccination. Of note, those with baseline antibody concentrations below the lower limit of detection had lower antibody responses compared to those with higher antibody levels at baseline. Infants born to vaccinated mothers had significantly increased antibody levels at birth, which persisted above placebo group levels at least 3 months after birth. Antibody concentrations decreased after birth and by day 91 were 22–25% of the levels measured at birth but were still 5–8.5 fold higher than those observed in the placebo group. There was only one (2%) preterm infant in the vaccine group, and there was no clear relationship between time from vaccination to delivery and maternal or neonatal antibody concentrations at birth for any of the serotypes. GBS-specific antibody ratios between vaccinated mother and infant (calculated as the paired ratio between the GBS-specific antibody concentration measured in the cord blood of the neonate to those measured in maternal sera at birth) ranged from 0.68 to 0.81 across the three serotypes (serotype Ia: 0.81, serotype Ib: 0.77, serotype III: 0.68). Currently, an extension study is underway to examine the safety and immunogenicity of a second dose of the trivalent vaccine administered in non-pregnant women after a time interval close to inter-pregnancy interval (NCT02690181).

### Safety and immunogenicity evidence among special populations

***HIV-infected mothers and their newborns.*** A non-randomized phase II open-label study using the trivalent (Ia, Ib, III) GBS polysaccharide-CRM conjugate vaccine was conducted in Malawi and South Africa among 270 pregnant women aged 18–40 years between 24–35 weeks gestation with or without HIV infection (NCT01412801) ^252^. There was no control group. Enrolment stratification ensured that about half of the HIV-infected women were in a low CD4 cell count category [50–350 cells/μL] or high CD4 cell count category [>350 cells/μL]. Results showed that immune response to vaccines as well as serotype-specific antibody levels in infants at birth were lower in HIV-infected mothers and their infants. In mothers, the fold change in antibody concentrations was higher for the HIV-uninfected group than the HIV-infected groups, and those with undetectable antibody levels at baseline had lower antibody concentrations post-vaccination compared to those with detectable antibody concentration at baseline. Transfer ratios (infant geometric mean antibody concentration in blood collected within 72 hours of birth divided by maternal geometric mean antibody concentration in blood collected at delivery) were similar across all three groups (0.49–0.72).

Rates of women reporting at least one solicited adverse reaction were highest in the HIV-uninfected group (67%), compared with HIV-infected women with a low CD4 cell count (44%) or high CD4 cell count (59%). Local reactions (most frequently injection site pain) were reported by 18–39% of women across the groups, and systematic reactions were reported by 40–59% of women (fatigue and headache were most frequent). Adverse events were reported by 74–78%, of which 7–23% were deemed to be caused by study vaccination. None of the reported serious adverse events (reported by 28–32% of women) or adverse events reported in infants (41–49%) were deemed to be caused by vaccination.

## Cost-effectiveness evaluation for low and middle income countries

GBS vaccine cost-effectiveness assessments may shed light on the potential investment case for GBS vaccines before phase III trials have been completed. Six analyses of GBS vaccine cost-effectiveness have been published to date, including four before the current era of GBS vaccine development ^297– 300^ and two recent analyses ^301, 302^.

The older studies evaluated cost-effectiveness in resource-rich settings (three in the United States and one in the United Kingdom). These documented the value of variants of screening- or risk-based intrapartum prophylaxis compared to ‘doing nothing’ and also assessed the potential value for a vaccine with assumed efficacy levels against GBS disease-causing serotypes, either as a maternal immunization strategy or as a vaccine delivered to adolescent females. The UK analysis ^300^ found that if a vaccine was available, the most cost-effective prevention strategy would include vaccination of all pregnant women, in combination with IAP for all preterm deliveries and a subset of term deliveries with risk factors (19% of all women treated). This study also emphasized the need for additional information on key model parameters.

Two more recently published cost-effectiveness analyses ^301, 302^ focused on the conjugate trivalent vaccine (serotypes Ia, Ib, III) in clinical development at the time, assuming a single dose of GBS vaccine would be recommended during each pregnancy. The Oster analysis evaluated the addition of universal vaccination of pregnant women to the screening-based IAP strategy in the United States. Assuming a vaccine cost of $100 per dose and75% vaccine efficacy against included serotypes among term deliveries and a reduced efficacy among preterm deliveries, this analysis found that the cost-effectiveness of maternal immunization may be comparable to other recently approved vaccines in the United States. A CDC-sponsored cost-effectiveness analysis for the United States is in progress, with results anticipated in late 2016.

The Kim analysis focused on the upper middle-income country of South Africa. This decision-analytic model simulated the natural history of GBS transmission from mothers to infants and compared four strategies: do nothing, risk factor-based IAP, maternal GBS vaccination, and vaccination plus risk factor-based IAP. National and hospital-based GBS prevention policies in South Africa are consistent with variants of the risk factor-based IAP approach, although group of women eligible is quite narrow and implementation is limited. This analysis assumed a vaccine price per dose of 10–30 U.S. dollars (USD) and vaccine efficacy against included serotypes of 50–90% among term infants with a reduction among preterm infants. The most influential parameters in one-way sensitivity analyses were vaccine price per dose and early onset GBS disease incidence. This analysis concluded that maternal immunization would lead to important reductions in the burden of infant GBS disease and be considered very cost-effective (range 416–3,545 in 2010 USD/DALY averted comparing vaccination to doing nothing; range 461–5,491 2010 USD/DALY averted comparing vaccination to risk factor-based IAP). Notably, vaccination plus risk factor-based IAP was more effective and consistently very cost effective. Risk factor based IAP alone was also very cost effective but prevented only a small burden of infant GBS disease.

Sinha and team are also in the process of conducting a GBS vaccine cost-effectiveness analysis for GAVI-eligible low-income sub-Saharan African countries. Thirty seven countries in the region were clustered into four groups based on 24 measures of economic development, general health resources, and past success in public health programs. A decision-analytic model was built to compare a natural history arm (‘do nothing’) with maternal immunization as part of antenatal care. Risk factor-based IAP was not included in this assessment due to expert opinion that this was not feasible for these low income birth settings. Results are expected in late 2016.

## Mathematical modeling related to GBS vaccines

Mathematical models, can inform decision-making related to vaccine development and implementation in several ways. For example, disease transmission models can shed light on the impact of varying key aspects of vaccine delivery such as age at vaccination, dosing schedules and method of delivery ( *e.g.*, vaccine campaigns versus incorporation into routine schedules). Models can also clarify the potential impact of a vaccine on unvaccinated members of the population (herd immunity) and predict potential unintended consequences of vaccine introduction such as an increased age at first infection, or the potential for replacement disease due to strains not included in the vaccine candidate. Finally, mathematical models can often highlight influential parameters where there would be value in a strengthened evidence base to allow for more accurate estimates.

In the context of maternal immunization for GBS, mathematical modeling to date is extremely limited. Some of the cost-effectiveness models developed have included a natural history arm that estimates disease burden based on a variety of maternal risk factors ^301^. A non-dynamic compartmental model that estimates GBS-related outcomes based on maternal GBS colonization and risk of neonatal disease is under development as part of a global GBS disease burden estimation activity led by the London School of Hygiene and Tropical Medicine. It is possible that models could prove useful to better understand the impact of maternal vaccination timing on the preventable portion of newborn disease, particularly since earlier vaccination may offer protection to later preterm deliveries, depending on antibody transfer ratios and decay rates. If conjugate GBS vaccines have an appreciable effect on reducing acquisition of GBS colonization with vaccine-included serotypes, models may also help assessing the consequences of reduced exposure of the newborn to GBS. Models may also help predict the impact of maternal immunization across LMIC settings with different prevalence of maternal HIV infection, levels of home vs facility deliveries, and optimal window for vaccination considering also antenatal care seeking behaviors. Models could contribute to the understanding of the potential impact of GBS vaccination on the burden of GBS-related stillbirth and preterm delivery.

## Considerations about options to generate pivotal licensure data

The present document intends to provide an overview of available options and a framework for future reflection and should not be interpreted as guidance or recommendations.

### Trial design options

Double-blind individually randomized controlled trial designs generate most robust data and minimize risks of bias. A relevant clinical endpoint ^288^ supportive of efficacy evaluation provides the most direct evidence of the potential health impact. However, low baseline disease incidence may lead to very large sample size requirements for vaccine efficacy evaluation. A potential alternative option may be to use immunologic correlates of protection as the primary endpoint ^76, 272^. Correlates of protection have been used for licensure in future generations or variants of a licensed product, or in instances where direct efficacy against disease is not readily feasible and correlates of protection are well-established. The evidence supporting recognition of a correlate of protection may be derived from an efficacy trial, which provides the opportunity for nested immunogenicity evaluations and detailed analysis of the relationship between immune and clinical endpoints. Alternatively, as in the case of GBS, immunological correlate of protection may be inferred from sero-epidemiological studies.

Correlates of protection have indeed been used for licensure of meningococcal, pneumococcal conjugate, and inactivated influenza vaccines. Group C meningococcal conjugate vaccine was licensed in the United Kingdom based on immunogenicity studies without efficacy data. These compared serum bactericidal assay titers induced by the experimental vaccine with a licensed serogroup C polysaccharide vaccine, which had an established evidence of efficacy and correlates of protection ^274, 303^. The 10-valent pneumococcal conjugate vaccine (PCV10) and the 13-valent pneumococcal conjugate vaccine (PCV13) were licensed based on non-inferiority trials compared against PCV7 using serological end-points ^304^. Use of immunogenicity bridging studies comparing new vaccine products with those with established clinical efficacy is an accepted licensure pathway for inactivated seasonal influenza vaccine ^305, 306^. If licensure is granted based on a primary immunogenicity endpoint, there may be a regulatory requirement for post-licensure evaluations of effectiveness against disease endpoints. The optimal design of post-licensure trials need careful considerations as the inclusion of a non-vaccinated study arm may be deemed ethically unacceptable. Alternative case control, cluster randomized or ecological studies are possible.

### Possible study endpoints

***Trial with a disease endpoint***

**GBS disease** GBS invasive disease in young infants would likely be viewed as a relevant primary efficacy endpoint (see Table 8) ^76^. Given that GBS-related stillbirths have similar pathophysiology as neonatal GBS disease (ascending infection from a colonized mother), using a composite disease endpoint that includes GBS-related stillbirths is a possibility, which could help reduce the study sample size. Subgroup analyses may be used to assess the influence of various maternal factors ( *e.g*., HIV infection, malaria, malnutrition, maternal age, multiparity) on protection. Factors that influence the extent of protection, such as when maternal vaccination occurs in relation to the birth of the child (allowing sufficient time for a maternal antibody response), the gestational age at birth (placental transfer will be less in those born prematurely) and the chronological age of the infant (antibody levels will wane over the first 2–3 months of life), may also need to be characterized ^76^. Analyses of vaccine serotype-specific efficacy and efficacy stratified by term vs. preterm, early-onset disease vs. late-onset disease, and serotype-specific efficacy could be conducted ^76^. Other endpoints of public health interest such as prevention of prematurity, stillbirths, hospitalization, and mortality could be considered but interpreted carefully in the context of multiple statistical testing and statistical power. See Table 8 for summary of candidate case definitions.

**Table 8.** Possible case definitions for phase III vaccine trial primary and secondary endpoints.

	Candidate case definition	Pros	Cons
Infant invasive GBS disease --Early-onset --Late-onset --Young infant	GBS isolated from a normally sterile site (e.g., blood, CSF) by culture in an infant with PSBI Onset of invasive GBS disease during days 0–6 of life Onset of invasive GBS disease during days 7–89 of life Onset invasive GBS disease during days 0–89 of life	• Gold standard clinical endpoint • GBS isolates can be used to further characterize the strains • Young infant: provides the largest number of cases • Early-onset: Pre-clinical and Phase II data suggest efficacy for this endpoint may be higher than for late- onset or a combined endpoint	• Low baseline rates • Requires high capture rate of ill babies, in particular on day 0 of life, for PSBI assessment • Requires specimen collection and access to qualified laboratory within a short period after case capture • Blood culture sensitivity may be low and varies depending on how specimen is collected and processed
Probable/possibleGBS sepsis	Clinical sepsis (see below) plus surface colonization with GBS and no other sepsis cause identified	• Higher baseline rates • Swab specimens may be easier to collect and process than sterile site specimens	• Low specificity for GBS disease
Clinical sepsis	PSBI: Any one of: not feeding well, convulsions, fast breathing [≥60 breaths/min], severe chest indrawing, fever [≥37.5°C], low body [<35.5°C] temperature, movement only when stimulated or no movement at all	• Very high baseline rates • LMIC are familiar with this definition • This definition links to global causes of death and verbal autopsy data	• Very low specificity for GBS disease
Premature birth	Birth at <37 weeks’ gestation	Provides a standardized definition that has been widely used	• An accurate estimate of gestational age is required. • Not GBS-specific and the contribution of GBS is likely low
Stillbirth	Baby born with no signs of life at or after 28 weeks’ gestation	A definition currently suggested by the WHO for international comparison.	• An accurate estimate of gestational age is required. • Definition is not specific for GBS and GBS only accounts for a limited portion of stillbirths
GBS-related stillbirth	Stillbirth (as defined above) with GBS confirmed by autopsy or by culture from a normally sterile site (e.g., fetal heart, CSF)	May decrease the sample size if used as part of a composite disease end point.	Autopsy may not be culturally acceptable in certain communities. Stillbirth will need to occur at facilities that have the capacity to perform autopsy.
Maternal and/or young infant GBS colonization	Maternal: Positive GBS vaginal and/or rectal culture Young infant: Positive GBS culture from surface swabs (most typically umbilicus, nares, outer ear)	• Moderate baseline rates • Low complexity with regards to specimen collection and processing • Yields isolates for further strain characterization • Sheds light on vaccine impact on carriage	• Requires sites to have a standardized specimen collection and processing procedure. • Knowledge of colonization status before delivery may necessitate IAP. • Indirect measurement of disease endpoint: a lack of reduction of colonization may not mean lack of protection against invasive infant disease

CSF: cerebrospinal fluid, GBS: group *B* streptococcus, IAP: intrapartum antibiotic prophylaxis, LMIC: low- and middle-income countries, PSBI: probable severe bacterial infection, WHO: World Health Organization

**Colonization** Newborn GBS colonization or exposure from colonized mothers is a precursor to GBS disease. The demonstration of vaccine efficacy against maternal and newborn colonization may argue for a protective effect of GBS vaccination. If vaccination reduces vaginal GBS colonization with the targeted invasive strains at the time of delivery, the risk of developing early-onset disease and potentially late-onset disease by strains targeted by the vaccine would likely decrease ^76^. However, other factors may play a role, as only a small proportion of colonized neonates develop disease. Further considerations on case definitions are provided in Table 8.

***Trial with immunologic correlates of protection.*** For glycoconjugate GBS vaccines, evidence from immune-epidemiological studies suggest that maternally-transmitted, functional IgG antibodies against GBS capsular polysaccharides, as measured by a quality-assured opsonophagocytic assay in serum from neonates and/or young infants may constitute a candidate substitute endpoint (see immune correlates of protection section). Further evidence is needed to evaluate the possible role for immune markers of protection induced by protein vaccine candidate in the licensure pathway.

**Considerations for licensure based on immune markers** While associations between antibody concentrations and risk of disease have been observed, the strength and nature of these associations require further investigation and continued assay standardization efforts. Several analytical frameworks for validating immune markers as substitute endpoints for protection against clinical disease have been developed ^274^. The Prentice Criteria (see Box 2), originally designed for randomized-controlled trial data, but extended by others to observational designs ^274^, can be used to evaluate potential substitute endpoints.

Box 2. **Prentice Criteria for validation of a surrogate endpoint ^274, 328^**

1. Protection against the clinical endpoint is significantly related to having received the vaccine
2. The substitute endpoint (immune marker) is significantly related to vaccination status
3. The substitute endpoint is significantly related to protections against the clinical endpoint
4. The full effect of the vaccine on the frequency of the clinical endpoint is explained by the substitute endpoint, as it lies on the sole causal pathway

The evidence base to evaluate whether a substitute endpoint fulfils the Prentice Criteria would most typically come from a trial with a clinical disease endpoint and nested immune marker study. For a GBS candidate vaccine, evidence for these criteria may need to be gleaned from a range of experimental and observational studies. The first criterion, that protection against the clinical endpoint is significantly associated with vaccine receipt, may derive from animal challenge studies. Evidence for the second criterion (the immune marker is significantly related to vaccination status) would likely derive from phase II studies. Evidence for the third (the substitute endpoint is significantly related to protection against the clinical endpoint) would likely derive from sero-epidemiological observational cohort and case-control studies. Evidence for the fourth criterion may come largely from existing knowledge about immune response and protection among young infants in the first 3 months of life.

The Prentice Criteria are not the only approach to evaluation of a substitute endpoint. The Qin framework ^307^ can also be applied. This framework distinguishes associations between immune markers and clinical disease endpoints into three classes, and within these also offers more options for causal inference frameworks that can be applied. This framework also highlights whether a substitute endpoint is specific to a single population (the data derived just from one population) or whether it is general (meaning it has been observed in multiple populations).

### Endpoint case definitions

Isolation of GBS from a normally sterile site, such as blood or CSF in an infant with possible sepsis or meningitis, is a widely used definition for young infant invasive GBS disease ^22, 24, 32, 37, 38, 46, 146, 147^. GBS isolation by culture is considered the reference standard. Automated culture methods yield higher detection rates compared to manual culture methods ^105, 308^; minimizing time between collection and inoculation of blood culture bottles, using pediatric bottles for young infants, and maximizing blood volumes are important for optimal results ^105^. For GBS meningitis, in addition to positive CSF culture, case definitions have included detection of GBS antigens in CSF ( *e.g.*, latex agglutination) ^24, 46^, detection by PCR ^46^, and GBS positive blood culture with CSF findings consistent with meningitis ^22, 46, 47^. As described, onset of disease during days 0–2 or 0–6 of life is commonly used for early-onset disease and onset during days 7–89 is used for late-onset disease ^3, 24, 32, 38, 46, 147^. Due to challenges in surveillance for invasive disease, some young infant studies have developed case definitions for probable GBS infection capturing infants with clinical sepsis and surface colonization with GBS ^309– 311^. Because surface colonization of young infants can be common, however, such definitions have limited specificity. Recently, some studies have used PCR on whole blood in addition to blood culture ^95, 312^. This can enhance detection but blood samples from healthy controls provide an important context: a low percentage of healthy controls have been documented with positive PCR on blood in both South Africa and South Asia (SANISA and ANISA unpublished studies). Another option for newborn disease is clinical sepsis. Several definitions have been used. PSBI, as defined by IMCI ^103^ is sensitive but not specific: the sensitivity is estimated to be 85% and the specificity of 75% based on an experienced pediatrician’s assessment ^101^. South Africa has used a more specific definition including both clinical and laboratory signs ^15^. Use of chest X-rays may be considered if pneumonia is one of the outcomes of interest. Candidate case definitions are summarized in Table 8.

### Sample size

The number of young infant GBS cases at single institutions is relatively small, depending on the number of annual deliveries and the disease incidence rate. A trial with a disease endpoint would likely necessitate a multi-center trial. An efficacy trial conducted in settings where standards of care include screening-guided IAP would lead to very large sample size requirements. If acceptable, trial conduct in a high incidence setting where screening-based IAP is not implemented as standard of care would reduce the sample size requirement. Adequate infection risk management in study participants would need to be discussed with relevant authorities and institutional review boards (IRBs), in consideration of local recommendations and WHO recommendations. Acceptability may be higher if favorable safety has already been established in a significant number of individuals. At a site with an incidence of 2.0 per 1000 live births for neonatal GBS disease <90 days of age, approximately 34–44,000 pregnant women will need to be enrolled (assuming that 75–85% of neonatal GBS disease are caused by vaccine serotypes, 70–80% are eligible, and 90% power to detect vaccine efficacy of 75% against vaccine serotypes), whereas >100,000 pregnant women will be needed in countries such as Europe and North America where IAP has reduced the incidence of early-onset disease to markedly less than 1 per 1000 live births ^76^ ( Table 9. For conjugate vaccines this number will vary depending on the GBS serotypes contained in the candidate vaccine and the serotype distribution at the study site. A licensure trial based on an established immune correlate of protection would require a smaller sample size and the total pre-licensure exposed population would likely be determined by safety characterization requirements. Considerations for safety evaluation are described in “ safety considerations” and “ regulatory considerations and potential licensure pathways for low- and middle-income countries”.

**Table 9.** Estimated sample size for a phase III randomized controlled trial using a clinical endpoint of invasive neonatal GBS disease.

Incidence rate of invasive neonatal GBS disease (per 1,000 live births)	Estimated sample size*
0.25 (Early-onset disease, U.S., 2014) ^1^	275,000–356,000
0.45 (Early-onset disease, pooled estimate for Europe) ^2^	153,000–198,000
0.73 (Late-onset disease, pooled estimate for sub-Saharan Africa) ^3^	94,000–122,000
1.3 (Early-onset disease, pooled estimate for sub-Saharan Africa) ^3^	53,000–68,000
2.7 (Overall GBS incidence during ages 0–90 days, South Africa, 2004–2008) ^4^	25,000–33,000

* Assuming 90% power to detect 75% vaccine efficacy, 75–85% of disease are vaccine types, 70–80% of approached participants are eligible per protocol 1. Active Bacterial Core Surveillance, 2014 2. Edmond *et al.* 2012 ^3^ 3. Sinha *et al.* 2016 ^51^ 4. Cutland *et al.* 2015 ^47^ GBS : group B *Streptococcus*, U.S.: United States

### Safety considerations

Evaluation of safety for a vaccine that will be specifically approved for use in pregnant women is unique given that: (1) the safety of both the mother and the fetus/child will need to be considered, and (2) complications of pregnancy may occur even in pregnancies considered as “low risk” regardless of the vaccination status ^288^. Therefore, the relative risk of common adverse pregnancy outcomes in the study population should be determined. Sample sizes must be adequate considering baseline incidence of adverse pregnancy outcomes and may not be finalized until phase II safety data are available. Detection of rare adverse outcomes require large sample size. Baseline studies can be useful to determine sample size needs, which should be discussed in advance with regulators.

One of the challenges in assessing safety of maternal immunization has been a lack of standard definitions for maternal immunization adverse events ^313^. In 2014 WHO held a consultation jointly with the Brighton Collaboration to facilitate harmonization of existing key terms to support monitoring of vaccine safety in pregnant women and newborn children ^314^. Key terms were chosen for discussion based on (1) frequency, (2) severity of health outcome, (3) public health relevance, and (4) measurability in different settings. The Global Alignment of Immunization Safety Assessment in pregnancy (GAIA) established working groups to review the evidence from the WHO-Brighton Collaboration landscape analysis, and has developed a set of interim case definitions according to the Brighton Collaboration process and format ^315^. As of September 2015, the working group has developed 10 case definitions currently undergoing peer-review ^316^.

### Ethical and standard of care considerations

There is no regulatory or ethical prohibition on studies in pregnancy ^317– 319^. However, the concept of maternal vaccination, which may potentially pose harm to both the mother and the infant, may not be well received in countries where uptake of vaccines currently recommended for pregnant women by WHO is low ^320^. If a randomized-controlled study is designed, an important consideration is whether the control group should receive another vaccine that is currently recommended by WHO rather than placebo ^76^.

Controversies exist surrounding whether trials in low- and middle-income countries with a high burden of GBS disease should offer universal screening and IAP to their participants, the worldwide “best available” standard of care ^321^. Arguments against this have been presented: provision of care that is not sustainable at the study site could produce results that are more generalizable in higher-income countries and have little social value for the host community ^321^. Additionally, provision of a standard of care normally not available could coerce pregnant women into trial participation. Authors have suggested that study sites should adhere to local recommendations, in consideration of WHO guidelines ^100, 102, 116^. The acceptability of a trial under local standards of care may be dependent on the benefit risk assessment and the available safety data on the candidate vaccine. Whatever the approach, GCP trials should be authorized and under oversight by local IRBs and recognized authorities, with participant agreement documented through an informed consent process.

### Considerations about research center characteristics in low- and middle-income countries

Phase III studies with clinical outcomes as endpoints would likely need to be conducted in geographical locations with a high burden of neonatal invasive GBS disease ( Table 9), which are likely to be in LMIC. Important trial site characteristics are reviewed in Table 10. Important aspects include the presence of experienced clinical-trialists and established Ethics Review Committees (ERC) and Regulatory Authorities (RA) oversight, to ensure the highest compliance with Good Clinical Practice standards ^76^; availability of clinical and laboratory infrastructure for optimal capture of PSBI cases for specimen collection, processing, and identification of GBS from collected specimens ^105^; proportion of home deliveries; access to care supportive of rapid clinical sepsis diagnosis and collection of appropriate specimens close to disease onset ^76^; capacity to assess gestational age, provide sufficient medical care and to identify and respond to adverse events in both vaccinated pregnant women and their newborn infants ^76^. Clinical management study algorithms can support standardized collection of safety events and endpoints of interest according to defined case definitions.

**Table 10.** Important characteristics for study sites for GBS vaccine trials.

Characteristic	Regardless of endpoint	Specific to disease endpoint	Specific to immunogenicity endpoint
Disease burden	- Evidence of GBS disease as a contributor to neonatal sepsis - country level interest in GBS vaccine	- High baseline rate of invasive disease (see Table 8, Table 9) despite implementation of local standard of care Knowledge of circulating disease-causing serotypes	- Prior information on maternal colonization rates
Location/population	- Generalizability to LMIC in the primary regions targeted for vaccine - General acceptance in the population of vaccination and vaccines during pregnancy - Oversight by appropriate ERC/RA	- Delivery hospital serving a defined ANC catchment and a large annual number of deliveries to contribute to achievement of sample size targets	- Acceptance in the population of blood draws from mothers and young infants
Laboratory capacity	- Reliable freezers for appropriate storage of specimens related to endpoints - Ability and willingness to ship specimens out of country for further analysis - Capacity to process colonization swabs for GBS isolation - Familiarity/training in GCLP	- Strong onsite 24/7 blood and CSF culture capability at the labor and delivery hospital and primary admission location for ill newborns - Availability of automated blood culture machines, appropriate specimen collection bottles, and proficiency at bacterial isolation and GBS identification. - Capacity to perform serotyping by conventional methods or PCR; MLST; whole genome sequencing; antimicrobial susceptibility testing (or ability to ship sample to reference laboratory)	- Laboratory capacity for serum separation and aliquoting - Ability to ship specimens to designated reference laboratory (or ability to perform immunogenicity assays onsite)
Recruitment, enrollment and maternal vaccination	- Ability to reach pregnant women in the second trimester of pregnancy and to predict reliably where they will deliver - Ability to recruit, screen and consent pregnant women according to GCP - Ability to consent a high proportion of eligible women for enrollment in the trial - Ability to integrate vaccination and specimen collection (blood or swabs) into ANC visits - Ability for reliable estimation of gestational age	- Ability to reach a large portion of pregnant women in the delivery catchment, to allow for achievement of sample size targets	-
Birth capture	- Predesignated clinical facility for delivery and study staff on site to capture clinical, safety and endpoint information or specimens	- A medical records system that allows for capture of salient labor and delivery information (for example if IAP was administered) that may affect endpoint interpretation	- Ability to capture cord blood from a high proportion of enrolled delivering women
Endpoint capture	- A limited number of defined locations where study participant deliveries and maternal postpartum or young infant hospital admissions will take place	- 24/7 availability of trial staff or trained physicians on study procedures - An already established clinical practice of performing septic workups when babies fulfil IMCI criteria for possible serious bacterial infection - Capacity to collect lumbar punctures from newborns meeting possible meningitis criteria - Capacity and supplies for sterile collection of appropriate specimens upon onset of illness and where possible before administration of antibiotic therapy - Capacity for real-time investigation and specimen collection from stillbirths - Requirement for chest X-rays capacity to be considered	- Ability to capture repeated blood draws from the mother and the newborn/young infant at pre-designated intervals
Safety monitoring	- Capacity to follow up enrolled women before and after delivery for required time periods (six months or more) - Capacity to capture pregnancy and birth outcomes including clinical status at birth and presence of congenital anomalies - Capacity to follow newborns for adverse/serious adverse events for the required time period (six months or more) - Capacity to capture newborn developmental measures in addition to illness events	-	-
Local standards of care	- Antenatal, labor and delivery, postpartum and newborn standard of care policies consistent with local recommendations/WHO guidelines for local settings - Standards of care that will be supportive for generalization to other settings	-	-
Prior trial experience	- Prior experience with GCP and GCLP - Prior experience with clinical trials, ideally in pregnant women - Track record of ability to implement study protocols as part of a multi-center trial - Prior experience with vaccine studies - Prior experience with safety monitoring for Phase II and/or Phase III trials - Strong relationship with community and experience with social mobilization - Strong relationships with clinical staff in pediatric, microbiology and obstetric departments	- Prior experience with invasive GBS disease surveillance - Prior experience with large-scale studies of delivering women and newborns	- Prior experience with blood collection, especially from newborns -

ANC: antenatal care, CSF: cerebrospinal fluid, GBS: group B *Streptococcus*, GCP: Good Clinical Practice, GCLP: Good Clinical Laboratory Practice, ERC: ethics review committee, IAP: intrapartum antibiotic prophylaxis, IMCI: integrated management of childhood illness, LMIC: low- and middle-income country, MLST: multilocus sequence typing, PCR: polymerase chain reaction, RA; regulatory authority

***Potential challenges.*** Several review articles have summarized challenges in conducting studies that involve pregnant women in low- and middle-income countries ^320, 322^. Reaching women during the early stages of their pregnancy may be challenging in societies where women are reluctant about revealing their pregnancy early ^322^, and may miss the window of enrollment and vaccine administration. In addition, accurate estimation of gestational age, which is important in assessing pregnancy outcomes ( *e.g.*, preterm), is often a challenge in resource-limited settings. Measurements that are typically used, such as based on last menstrual period or measurement of fundal height, often do not provide consistent results. It is important for the participants to deliver their infants at predictable locations affiliated with the study and to be able to follow through on the follow-up visits to assess study-specific adverse events. However, this can be challenging in settings where regular follow-up visit after delivery is not customary.

## Regulatory considerations and potential licensure pathways for low- and middle- income countries

The regulatory considerations for products seeking an indication for use in pregnant women differ between already-licensed products and new products seeking licensure expressly for use among pregnant women. While there are several examples of already-licensed products with public health recommendations for use during pregnancy, there are no products yet that have achieved licensure for the specific indication of use during pregnancy. Respiratory syncytial virus (RSV) vaccines may represent the first pathogen class of vaccines that gain an initial indication for immunization of pregnant women as at least one RSV vaccine is ahead of GBS vaccines in their development timelines. Early dialogue between vaccine developers and regulators can play a particularly important role for maternal immunization product development. Major regulatory authorities, as well as the Council for International Organizations of Medical Sciences (CIOSM) in collaboration with WHO, have agreed that pregnant women should be presumed eligible for participation in research studies (CIOMS Guideline 17) ^323^, and that this applies also to vaccines intended to protect primarily the offspring.

Licensure in the United States and Europe can be requested through due Food and Drug Administration (FDA) and European Medicine Agency (EMA) processes respectively. The Article 58 pathway provides a collaborative review framework between the EMA and WHO, for products not intended to be used in Europe. Submissions can be done as specified by relevant national regulatory authorities in LMICs. The African Vaccine Regulatory Forum (AVAREF) is a collaborative forum of regulators from different African countries, constituted to enable information sharing between African NRAs. The Developing Country Vaccine Regulators’ Network (DCVRN) may also facilitate steps in regulatory processes in LMICs that are members.

Regulatory considerations from the FDA on the clinical development of vaccines indicated for use in pregnancy have been presented elsewhere ^288^. In addition to evidence to support safety and effectiveness claims, maternal immunization submission packages may need to include information on potential immune interference in the infant, due to the transfer of maternal antibodies to the vaccine antigen or to carrier proteins that may share epitopes with carriers used in the infant vaccine series. The role of immunological correlates of protection will need to be clarified, especially with regards to the primary licensure. Relevant quality-assured immunogenicity endpoints may also be used to bridge across populations, for instance when considering generalizability across LMIC or between LMIC and resource-rich settings.

Safety evaluations would be conducted considering vaccine effects on both pregnant women and their infants, taking into account background rates of common pregnancy complications ( *e.g*., pre-eclampsia, miscarriage/spontaneous abortion, stillbirth, preterm delivery). Pregnancy and neonatal outcomes, serious adverse events, new onset maternal medical conditions. The duration of safety follow-up for pregnant women and for newborns needs to be determined. Phase II data may be needed for optimal determination of Phase III sample size requirement for safety evaluation. Multiple factors including accumulated safety data associated with the product to date, safety signals, and the overall benefit to risk ratio assessment would likely be taken into account ^324^. See previous section for considerations on safety in phase III trials. Lastly, the need for post-approval investigations should be reflected on. To maximize chances of success for a candidate vaccine, vaccine developers should plan ahead to overcome potential post-approval obstacles ^325^. Target product profiles (TPP) list desirable characteristics, features, and attributes of a candidate vaccine, and have been long used by biotechnological and pharmaceutical companies ^326^.

## Areas for future research

We briefly summarize some of the leading scientific gaps relevant to GBS vaccine development and areas for future research based on the section topics addressed in this briefing document ( Table 11).

**Table 11.** Gaps and areas for future research.

Briefing document section	Gaps and areas for future research
Diseases and sequelae caused by GBS and population at risk	Young infant disease epidemiology • Preterm contribution to young infant disease burden, particularly in low-income countries (LIC) where preterm survival may be lower • Late-onset disease epidemiology in LMIC ○ Understanding how this differs from that described in high-income countries (HIC) (proportion preterm, ratio of early to late-onset disease, median age of onset, role of maternal HIV infection) • Role of SES, urbanization, and birth location (facility type, home vs facility) in GBS disease risk
GBS disease burden and serotype distribution	Young infant disease burden • Relationship between maternal colonization and young infant disease burden and serotype distribution • More information on burden from South Asia, Eastern Mediterranean Region, and South America • Sub-Saharan Africa: representation beyond southern Africa ○ Areas with low maternal HIV ○ LIC • More evidence on disease burden from other high mortality regions • Limited serotype data from LMIC, especially LIC Pregnancy-associated disease burden • Limited information from LMIC Stillbirth burden • Need for a standardized definition • Causes of stillbirth are not routinely collected in many countries • Challenges in obtaining appropriate specimens Non-pregnant adults • Limited information from LMIC
Diagnosis and treatment of GBS disease	GBS disease diagnosis • Diagnostic tests that are more sensitive than blood/CSF cultures, with high specificity (not positive in healthy infants) Stillbirth • An understanding of the right specimen criteria or tests to distinguish presence of GBS (colonization) from causal role of GBS in fetal demise See also Considerations for a phase III trial on case definitions and standards of care
Prevention of perinatal GBS disease through intrapartum antibiotic prophylaxis	Limited information on the feasibility and challenges in IAP implementation in LMIC (see also section Considerations for a phase III trial on standards of care)
GBS vaccine development	Number and timing of maternal vaccination, considering successive pregnancies • In relation to transplacental transfer of maternal antibodies • In relation to optimizing protection against young infant disease GBS vaccine antigen that would confer broader protection • common protein antigen • higher-valent glycoconjugate vaccine Defining dose level and options for adjuvantation if needed Immune correlates of protection • Need larger sero-epidemiologic evidence base that includes diversity in patient populations (e.g., LIC, preterm, mothers with underlying conditions) Assay standardization, qualification, validation • Role of functional antibodies
Cost-effectiveness evaluation	Limited assessment for LIC (one for sub-Saharan Africa in progress) Further define needs and opportunities in HIC and GBS vaccination outside pregnancy
Mathematical modeling related to GBS vaccines	In general, only a limited number of mathematical modeling conducted to date. Mathematical modeling on the following might be helpful: • Impact of maternal vaccination timing on the preventable portion of young infant disease • Predicting vaccine impact if vaccine reduces acquisition of GBS colonization with vaccine-included serotypes • Predicting the potential impact of maternal immunization across LMIC settings with different prevalence in underlying conditions (e.g., HIV) • Potential impact on GBS-related stillbirth and preterm delivery
Considerations for a phase III trial	Evaluation of validity of candidate substitute endpoints Study endpoints and case definitions • Development of agreed case definitions for disease endpoints, standard data collection methodologies • Define the role of colonization studies, consider possibility of strain replacement Define sample size requirements for safety Acceptability of local standards of care in context of a trial
Regulatory considerations ad potential licensure pathways for low- and middle-income countries	More clear indications from regulators on acceptable licensure pathways for maternal GBS vaccine, particularly with regards to use of substitute endpoints for primary licensure, and with regards to generalizability across the different settings where vaccine maybe used

CSF: cerebrospinal fluid, GBS: group B *Streptococcus*, HIV: human immunodeficiency virus, HIC: high-income countries, IAP: intrapartum antibiotic prophylaxis, LIC: low-income countries, LMIC: low- and middle-income countries, SES: socioeconomic status
