# Supplementary material for: Group B Streptococcus vaccine development: present status and future considerations, with emphasis on perspectives for low and middle income countries
Source: F1000Res. 2016 Sep 22;5:2355. [Version 1] doi: 10.12688/f1000research.9363.1 (PMC5070600; doi:10.12688/f1000research.9363.1)
Supplement: Peer review history

# Peer review: Referee response for version 1

**DOI:** 10.5256/f1000research.10082.r16507

**Published:** 2016-10-13

**Authors:** Ronald Francis Lamont

**Affiliations:** 1 Division of Surgery, Northwick Park Institute for Medical Research Campus, University College London, London, UK

Having personally published earlier this year on GBS vaccination ^1^, this is the most comprehensive review of GBS vaccination I have seen. Quite appropriately, the authors have extended the review beyond early onset GBS disease to include late onset GBS disease and GBS disease in non-pregnant adults.

 Unlike many reviews they have focused on low and middle-income countries (LMIC) where the burden of disease is at its greatest whereas most of the available data are from resource rich countries.

 In addition, the review covers other important topics often omitted from other overviews such as the increased risk of neonatal GBS disease in HIV-positive mothers, the use of protein based vaccines, reverse vaccinology and the necessary use of surrogate endpoints in vaccine trials.

 Importantly, they have addressed the problem that logistically, because of the estimated sample size for a phase III randomised controlled trial using a clinical endpoint of invasive neonatal disease, the different burden of disease across geographic regions dictates that trials may have to be performed in LMICs. This has its own problems, not the least of which is the acceptability of local standards of care in the context of such a trial.

 Finally, a minor criticism would be the omission of a discussion on the association between black race and GBS rectal colonisation, which is particularly pertinent bearing in mind the burden of disease in Africa and in African American women in the USA. In addition, while the use is mentioned, the role of rapid detection of GBS carriage using automated PCR techniques that might decrease the unnecessary use of intrapartum antibiotic prophlyxis during labour merits some emphasis.

I have read this submission. I believe that I have an appropriate level of expertise to confirm that it is of an acceptable scientific standard.

## References

[1] KenchingtonALLamontRF: Group B streptococcal immunisation of pregnant women for the prevention of early and late onset Group B streptococcal infection of the neonate as well as adult disease. *Expert Rev Vaccines*.2016; 10.1080/14760584.2016.1209113 1-11 10.1080/14760584.2016.1209113 27385362

# Peer review: Referee response for version 1

**DOI:** 10.5256/f1000research.10082.r16511

**Published:** 2016-10-10

**Authors:** Laura Elizabeth Riley

**Affiliations:** 1 Department of Obstetrics and Gynecology, Massachusetts General Hospital, Boston, MA, USA

This is an excellent review article summarizing the status of GBS vaccine development for global use. This manuscript, in a logical and practical way, lays out the case for further development and testing of a GBS vaccine to be given to mothers primarily for the protection of their neonates.

 The burden of maternal and neonatal disease is thoroughly discussed and highlights the paucity of epidemiological data in many resource limited settings. This is the critical data needed to determine the contribution of GBS to overall neonatal morbidity and mortality in low and middle income countries where a candidate vaccine is most likely to be beneficial.

 There is an excellent list and discussion of previous GBS vaccine trials and outcomes. Even more useful is the discussion of challenges going forward with GBS vaccine trials, including a review of potential trial endpoints, challenges of any maternal vaccination program, and potential ethical and regulatory hurdles.

I have read this submission. I believe that I have an appropriate level of expertise to confirm that it is of an acceptable scientific standard.
